# Designing flexible protein structures and sampling protein conformations with a unified model using vector quantization and diffusion

**DOI:** 10.1093/nsr/nwaf290

**Published:** 2025-07-16

**Authors:** Yufeng Liu, Linghui Chen, Quan Chen, Haiyan Liu

**Affiliations:** MOE Key Laboratory for Membraneless Organelles and Cellular Dynamics, School of Life Sciences, Division of Life Sciences and Medicine, Hefei National Research Center for Physical Sciences at the Microscale, University of Science and Technology of China, Hefei 230001, China; Anhui Basic Discipline Research Center of Artificial Intelligence Biotechnology and Synthetic Biology, University of Science and Technology of China, Hefei 230027, China; Oristruct Biotech Co., Ltd, Hefei 230026, China; MOE Key Laboratory for Membraneless Organelles and Cellular Dynamics, School of Life Sciences, Division of Life Sciences and Medicine, Hefei National Research Center for Physical Sciences at the Microscale, University of Science and Technology of China, Hefei 230001, China; Anhui Basic Discipline Research Center of Artificial Intelligence Biotechnology and Synthetic Biology, University of Science and Technology of China, Hefei 230027, China; MOE Key Laboratory for Membraneless Organelles and Cellular Dynamics, School of Life Sciences, Division of Life Sciences and Medicine, Hefei National Research Center for Physical Sciences at the Microscale, University of Science and Technology of China, Hefei 230001, China; Anhui Basic Discipline Research Center of Artificial Intelligence Biotechnology and Synthetic Biology, University of Science and Technology of China, Hefei 230027, China; School of Biomedical Engineering, Suzhou Institute for Advanced Research, University of Science and Technology of China, Suzhou 215127, China; Department of Ocean Big Data and Prediction, Laoshan Laboratory, Qingdao 266228, China

**Keywords:** AI for biology, protein structure prediction, protein design, machine learning

## Abstract

Conformational dynamics are often critical for protein functions. There is strong interest in deep learning models to predict the conformational distributions of proteins or design protein structures that can host rich conformational dynamics. Here we report PVQD (Protein Vector Quantization and Diffusion), a method using a vector-quantized auto-encoder to learn protein backbone latent representations and latent-space diffusion for backbone generation and for conformation sampling conditioned on native sequences. Comparisons show PVQD generates backbones with natural-like compositions of secondary structures, loop lengths, and domain sizes. In sampling conformations of natural proteins, PVQD better reproduces experimental structural variations in benchmark proteins than existing approaches. For K-Ras, KaiB, 4.1G CTD, and D-allose binding proteins, PVQD captures sequence-dependent effects on functional conformational dynamics. Thus, the latent space diffusion approach forms a valuable framework that can unify the prediction and design of protein structures with the capability of modeling conformational dynamics.

## INTRODUCTION

Recent advances in deep learning enable accurate end-to-end modeling of protein sequences (1D) and structures (3D) for structure prediction [1–3], inverse protein folding [4,5] and *de novo* backbone design [6–9]. Despite these developments, some important needs of the field are still unsatisfied with current models. Current deterministic 3D prediction methods fail to predict conformational variation [10–12]. While a subsampled MSA approach generates structural ensembles [13], it is unusable for MSA-independent predictors and still requires improvement [12,13]. Additionally, designing proteins with rich conformational dynamics, essential for catalytic/regulatory functions [14,15], remains challenging. Existing designed proteins verified in a wet lab are mostly rigid, comprising tightly packed secondary structures and short loops [6,7,9] and lacking features of conformational flexibility like natural enzymes or allosteric proteins.

These two needs could be concurrently met with an approach that can sample protein structures unconditionally or conditioned on sequence. Denoising diffusion probabilistic models (DDPMs), successful in image or speech generation [16,17], have been used in generating protein structures [6–9]. Current protein DDPMs use diffusion either in indirect, user-specified feature spaces (e.g. 2D distance maps) to produce restraints for 3D model building [18], or directly in 3D space [6–9]. Various *de novo* protein structures from direct-3D-diffusion have been validated by wet experiments [6–9]. However, these structures are often comprised of only a single, tightly packed core with short, idealized loops, indicating the lack of rich conformational dynamics, [7,9,19]. For structure prediction, several DDPM-based conformational sampling methods exist [20], but they lack the accuracy of state-of-the-art predictors, with the quality of the sampled conformational ensembles not matching those from MSA subsampling when compared against experimentally-measured structural fluctuations.

Beyond direct data-space or indirect user-specified-space diffusion, generative DDPMs performed in learned latent spaces [16,21,22], typically using auto-encoder frameworks [16,22–24], have been successfully applied in other fields [16,21,22,24,25]. A key advantage of diffusion in the latent space is the division of modeling the complex original data distribution between a DDPM (modeling a smoother, more weakly correlated distribution in the latent space) and a decoder (reconstructing high-frequency, intricately correlated structures in the original space). This approach is theoretically well-suited for modeling 3D protein structures, where physical plausibility depends on both smooth global features and high-frequency local details.

In this work, we implemented this approach for protein backbone modeling and developed PVQD (Protein Vector Quantization and Diffusion). Unlike previous protein DDPMs, PVQD diffuses in a latent representation space learned via a vector-quantized auto-encoder [26] instead of in original 3D space or user-specified feature spaces. As a generative model, PVQD can be applied for backbone design; using sequence as a diffusion condition enables structure prediction, thus unifying both structure design and structure prediction. We show that PVQD generates designable structures potentially hosting richer conformational dynamics than direct 3D diffusion models. In prediction, PVQD reproduces experimentally observed conformational variations of natural proteins with high correlations.

## RESULTS

### An overview of PVQD

**Figure 1.**
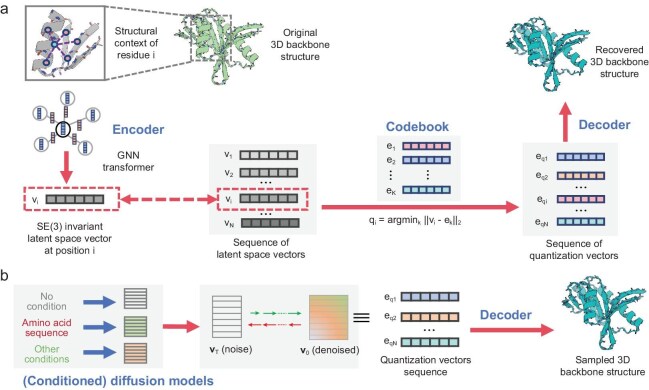
The overall architecture of PVQD. (a) The PVQD auto-encoder comprises an encoder, a codebook, and a decoder. The encoder transforms the 3D backbone structure into an array of SE(3)-invariant latent space vectors. These vectors are replaced by the closest quantization vectors contained in the codebook. The resulting array of quantization vectors is used by the decoder to best recover the original 3D backbone structure. (b) The PVQD generator performs denoising diffusion in the latent vector space to generate denoised arrays of quantization vectors, from which three-dimensional backbone structures are constructed through the codebook and decoder. The generator can be controlled by conditioners for various purposes. For structure prediction, the amino acid sequences are used as conditions.

The overall architecture of PVQD is depicted in Fig. 1. Details of the various network blocks are presented in the Methods section. The entire model comprises an auto-encoder with vector quantization and a generator using denoising diffusion.

The auto-encoder (Fig. 1a) was constructed to represent residue structural contexts as discrete codes via an encoder, a codebook, and a structure decoder. An SE(3)-invariant encoder embeds residue structures into a latent space, vector-quantized by the codebook [26] into structure codes. The structure decoder reconstructs the 3D structure from the quantized vectors.

For structure generation (Fig. 1b), PVQD employs Gaussian noise diffusion to model and to sample from the joint distribution of the array of the quantization vectors. The sampled vectors are decoded into 3D structures with the pre-trained decoder. Sequence-conditioned generation (for single-sequence-based structure prediction or conformation sampling) is achieved by embedding conditions during fine-tuning of the unconditioned diffusion network, following text-conditioned image generation approaches [16].

### The accuracy of the auto-encoder of PVQD

To assess if PVQD's auto-encoder can comprehensively represent and accurately reconstruct protein backbone structures, we compared three models. Two had distinct regularizations: a variational auto-encoder (VAE) [27] restraining the latent space toward standard normal distribution using Kullback–Leibler divergence loss; and a vector quantization auto-encoder (VQ-VAE) [26]. The third was a simple auto-encoder (AE) without latent space constraints. All models were trained identically on the same data until convergence of the reconstruction loss. Tested on unseen proteins, reconstruction accuracy was quantified with recRMSD (RMSD of atomic positions after superimposition using TMalign). The results are shown in Fig. S1A, with smaller recRMSDs indicating higher reconstruction accuracy.

All models achieved recRMSDs below 2.0 Å across protein sizes, indicating high backbone reconstruction accuracy. For proteins of <450 residues, AE, VAE and VQ-VAE exhibited comparable recRMSDs (all below 1.5 Å). However, for proteins of >550 residues, VQ-VAE had higher recRMSDs than AE or VAE. Yet only VQ-VAE produced latent distributions suitable for denoising diffusion (shown below). Thus, VQ-VAE was chosen to balance reconstruction accuracy and generation feasibility.

We observed that two VQ-VAE hyperparameters critically impact backbone reconstruction accuracy: codebook size (quantization vector count) and training sample cropping size. Larger codebook sizes reduced reconstruction error, plateauing at 8192 codebook sizes (Fig. S1B). Figure S1C shows that decoder fine-tuning with larger-crop data (without modifying encoder or codebook) further reduced reconstruction errors on larger proteins. Henceforth, PVQD's auto-encoder refers to VQ-VAE regularized by an 8192-vector codebook, with its decoder fine-tuned on structures cropped to 640 residues unless specified otherwise.

We also tested the auto-encoder's ability to reconstruct designed non-natural protein backbones using 63 SCUBA-D–generated structures [9], finding accurate reconstructions with recRMSDs below 0.8 Å (Fig. S1D). These structures exhibit low overall similarity to PDB structures (highest TM-scores below 0.5) and were confirmed as designable (self-consistent RMSDs or scRMSDs below 2.0 Å, where scRMSD compares a designed backbone to its AlphaFold2-predicted structure [2] based on ProteinMPNN-designed sequences [5]). To assess generalizability to untrained protein complexes, we evaluated 9276 non-redundant PDB complexes (resolutions higher than 4.0 Å). The auto-encoder achieved similar reconstruction performance for protein complexes and single-chain proteins (Fig. S1E), demonstrating generalizability to multi-chain structures.

To examine possible correlations between the quantized latent space vectors with commonly used structural features, we examined correlations between quantized latent space vectors and structural features by visualizing codebook vector distributions in 2D using t-distributed Stochastic Neighbor Embedding (t-SNE [28]), coloring points by structural features of assigned residues (averaged over non-redundant training proteins). Figure S1F colors points by averaged neighbor count (inter-Cα distance cutoff neighbors set to 15 Å), showing buried residues (more neighbors) generally separated from exposed residues (fewer neighbors) in latent space. However, no simple mapping relationship exists between latent codes and common structural features; residues sharing the same code do not necessarily share the same secondary structure type. Thus, the structure code of individual residues does not correspond to conventional residue-wise features. Instead, the array of codes of contiguous residues cooperatively encode the structural context, as demonstrated by Fig. S1G comparing the accuracy of residue-wise feature classification from individual codes versus from code arrays.

### Performance of PVQD in generating backbones without condition

We tested whether DDPM could model natural protein structure distributions in latent spaces from different auto-encoders. Using identical network architecture, we separately trained DDPM_VQ-VAE_, DDPM_VAE_ and DDPM_AE_ models in their respective latent spaces. We used each DDPM to unconditionally generate 100 backbones of 100 residues and evaluated the scRMSDs. Figure S1H shows DDPM_VQ-VAE_ achieved significantly lower scRMSDs than DDPM_VAE_ and DDPM_AE_, confirming the necessity of VQ-VAE for latent space regularization despite its reduced reconstruction accuracy. Thus, in the remaining study, we use the finally trained DDPM_VQ-VAE_ as the diffusion model in PVQD with linear configuration of noise schedule and 400 denoising steps (see Supplementary Notes for comparison on different configurations).

**Figure 2.**
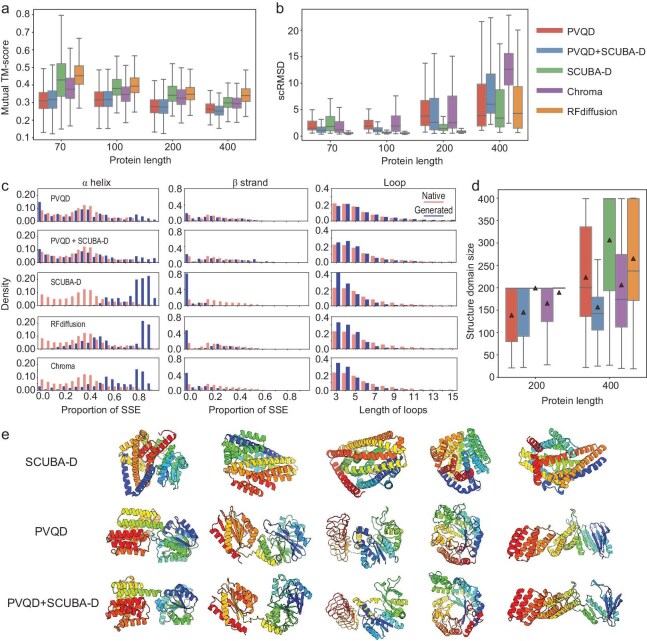
Computational analyses of protein structures unconditionally generated with PVQD and with other methods. (a) The distributions of the mutual TM-scores between the generated backbones of different sizes using different methods. (b) The distributions of the self-consistent RMSDs (scRMSDs), which are the RMSDs between the generated backbones and the structures predicted by ESMFold for amino acid sequences designed for the generated backbones. The predictions were performed on 8 ProteinMPNN-designed amino acid sequences for each backbone and the smallest RMSD values were used. (c) Histograms of the proportions of residues in the α helix state (left) and in the β strand state (middle), and the histograms of loop lengths (right) for the unconditionally generated backbones using different methods. For comparison, the corresponding histograms computed on the structures of the set of non-redundant natural proteins are also shown. (d) Distribution of the sizes of structural domains in the unconditionally generated backbones of 200 and 400 residues using different methods. (e) Examples of 400-residue backbones unconditionally generated with SCUBA-D (top), PVQD (middle) and PVQD + SCUBA-D (bottom). The boxplots show median, interquartile range, and minimum and maximum values excluding outliers (>1.5 times the interquartile range beyond the box). The red triangle inside each box refers to the mean value.

We compared the performance for unconditional backbone generation of PVQD against three other DDPM-based methods: SCUBA-D [9], Chroma [6] and RFdiffusion [7]. Unlike PVQD, these methods diffuse directly in a 3D structure space. We also tested PVQD + SCUBA-D, a hybrid method using PVQD to generate initial backbones which are then refined by SCUBA-D (see Fig. S2 for TM-score distributions between PVQD-generated and refined backbones, including eight examples). With each method, 400 backbones (each of 70–400 residues) were generated and evaluated in three aspects (Fig. 2a to 2d): backbone diversity measured via mutual TM-scores (Fig. 2a); designability assessed by scRMSD (Fig. 2b); and structural biases examined through secondary structure compositions, distributions of loop length (Fig. 2c) and distributions of structural domain size (Fig. 2d).

Figure 2a shows backbones generated from PVQD and PVQD + SCUBA-D exhibit lower mutual TM-scores (median values below 0.33 across lengths) than those generated by SCUBA-D (median 0.30–0.43), RFdiffusion (0.33–0.45) and Chroma (0.29–0.38). This indicates latent-space diffusion produces more diverse, sparsely distributed structures than direct 3D-space diffusion.

Figure 2b shows PVQD backbones exhibit scRMSDs comparable to Chroma but higher than SCUBA-D and RFdiffusion. PVQD, SCUBA-D and Chroma are freshly-trained DDPMs, while RFdiffusion fine-tunes a pre-trained RoseTTAFold model. Freshly-trained DDPMs may generate physically implausible features (thus higher scRMSDs) as standard DDPM loss recovers training data without penalizing out-of-distribution samples. While SCUBA-D mitigates this via adversarial losses [9], PVQD and Chroma do not. Nevertheless, substantial PVQD backbones are designable: using scRMSD below 2.5 Å for shorter backbones (of <300 residues) and below 3.5 Å for longer backbones (>300 residues) as thresholds, the fractions of designable backbones are 172/300 for shorter backbones and 48/100 for longer backbones. SCUBA-D refinement substantially improves designability for shorter backbones (212/300 designable) but degrades longer ones (11/100 designable), as refinement may cause the PVQD-generated structural domains to partition into several smaller domains, resulting in larger scRMSDs of the overall refined backbones. Geometric analyses (Table S1) show SCUBA-D refinement improves quality, reducing steric clashes (e.g. from 4.71 to 1.79 per residue for 100-residue backbones) and geometric distortions (from 5.71 to 3.96) and lowering the Rosetta energy (Fig. S3). This indicates PVQD + SCUBA-D balances designability and diversity by decoupling global conformation sampling (latent-space diffusion) from local structural optimization (3D-space refinement).

Existing studies have shown structural-space diffusion often generates α-helix–enriched, β-sheet–deficient structures [6,7], likely because contiguous helices form more readily than β-sheets, which require the coming together of disparate segments. Figure 2c confirms SCUBA-D, RFdiffusion and Chroma exhibit this bias, while PVQD and PVQD + SCUBA-D overcome it, producing secondary structure compositions similar to natural proteins. The PVQD models also yield loop length distributions resembling natural proteins (Fig. 2c), unlike the three direct-3D-diffusion methods which bias toward shorter loops. Notably, PVQD and PVQD + SCUBA-D generate backbones containing loops of more than 15 residues, which are rarely observed in structures generated with the other three models.

Natural proteins often contain multiple structural domains (common sizes: 100–200 residues), with inter-domain motions contributing to conformational dynamics. Figure 2d shows that 200-residue backbones from PVQD, Chroma and PVQD + SCUBA-D exhibit domain size diversity, whereas backbones from SCUBA-D and RFdiffusion typically form a single domain. For 400-residue backbones, SCUBA-D and RFdiffusion produce large domains of more than 300 residues, sizes infrequently observed in natural proteins. Figure 2e and Fig. S4 exemplify how PVQD and PVQD + SCUBA-D generate multi-domain structures with intermediate-sized domains, contrasting with other methods’ tendency toward generating single large domains.

With improvements in generating backbones with natural protein-like features including β-strand content and loop length/domain size distributions, we expect PVQD to generate designable protein structures that possess larger intrinsic flexibility and richer conformational dynamics relative to the rigid structures generated with current direct-3D-diffusion DDPM methods.

### Performance of PVQD in single-sequence-based structure prediction

**Figure 3.**
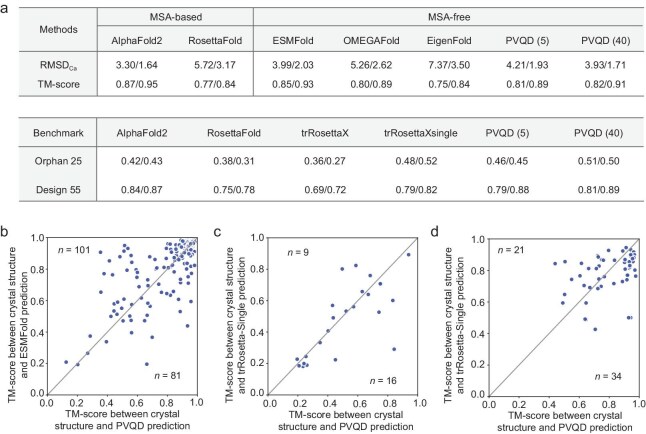
The performance of single structure prediction with PVQD. (a) The methods are compared on three datasets. The CAMEO2022 dataset is from Jing *et al.* [20], and the Orphan25 and Design55 dataset are from Wang *et al.* [31]. For each method, we compared the mean/median values of the RMSDs and TM-scores between the predictions and the native structures. For each target, PVQD was used to make five predictions (or 40 predictions for results shown in parentheses), and the prediction with the highest TM-score or the lowest RMSD to the native structure was selected. (b) Comparisons between ESMFold and PVQD (using 40 predictions per sequence) on the natural proteins in the CAMEO2022 dataset. ESMFold outperforms PVQD for 101 proteins in the upper triangle, and PVQD outperforms ESMFold for 81 targets in the lower triangle. (c) Comparisons between trRosettaX-single and PVQD (using 40 predictions per sequence) on the proteins in the Orphan25 dataset. The trRosettaX-single outperforms PVQD for 9 proteins in the upper triangle, and PVQD outperforms trRosettaX-single for 16 targets in the lower triangle. (d) The same as (c), but for the 55 human-designed proteins in the Design55 dataset. The trRosettaX-single outperforms PVQD for 21 proteins in the upper triangle, and PVQD outperforms trRosettaX-single for 34 targets in the lower triangle.

To use PVQD for single-sequence-based structure prediction, we tuned the DDPM in PVQD to take a given amino acid sequence as its condition. The resulting model was compared against seven other structure prediction methods on three benchmark sets (see Fig. 3 and Methods). Among the compared methods, AlphaFold2 [2], RosettaFold [1] and trRosettaX [29] are based on multiple sequence alignment (MSA), while ESMFold [3], OMEGAFold [30], EigenFold [20] and trRosettaX-single [31] are single-sequence-based. For PVQD, the results shown include both the best of 5 predictions and the best of 40 predictions for each target (here ‘best’ means the highest TM-score).

Figure 3a shows PVQD performance on CAMEO2022 targets (182 targets of <700 residues released between August 1, 2022 and October 31, 2022 [20]) is worse only than AlphaFold2 and slightly worse than ESMFold, but better than other methods. AlphaFold2 is MSA-based; ESMFold uses a larger language model (3B versus PVQD's 650M parameters). In Fig. 3b, the PVQD and ESMFold predictions are compared target-wise. PVQD outperforms ESMFold on 45% (81/182) of targets even with its smaller language model for representing the amino acid sequence.

For Orphan set proteins lacking MSA information, PVQD outperformed all MSA-based models and matched trRosettaX-single, a state-of-the-art single-sequence method [31]. Target-wise comparisons show PVQD outperformed trRosettaX-single on 64% (16/25) of targets (Fig. 3c), and the two methods are complementary to each other on some targets.

The benchmark items in the designed protein set also do not have MSA information but may contain much stronger structure signals in their sequences. PVQD again showed comparable performance as AlphaFold2 and trRosettaX-single for these proteins (Fig. 3a and 3d).

### Performance of PVQD in predicting multiple conformations of natural proteins

Inspired by the performance of PVQD in single-sequence-based structure prediction, we examined its ability to predict known multiple conformations using two existing benchmark sets [10,11], where each protein has at least two experimentally determined alternative structures. The apo-holo set contains 90 proteins undergoing ligand-induced conformational changes [10]; the fold-switching set includes 77 metamorphic proteins exhibiting varied secondary structures in specific regions [11]. For each protein, PVQD predicted 40 backbone structures, with the highest-TM-score prediction to each experimental conformation identified.

**Figure 4.**
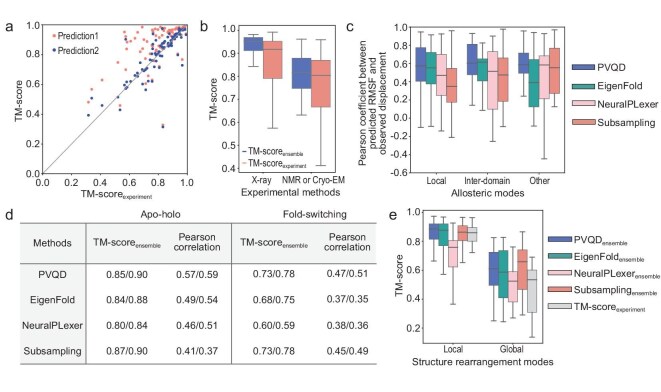
Comparisons of the PVQD-predicted structures with the experimental structures for two benchmark sets of proteins with multiple conformations. (a) For each protein in the apo-holo benchmark set, two experimental structures and 40-PVQD predicted structures were considered. For each experimental structure, the predicted structure of the highest TM-score was identified. The corresponding highest TM-scores versus the TM-scores between the experimental structures of the same proteins (i.e. TM-scores_experiment_) are shown in the scattering plot. For each protein, the point representing a higher prediction-versus-experiment TM-score is colored in salmon while the point representing a lower prediction-versus-experiment TM-score is colored in blue. (b) The distributions of the TM-scores_ensemble_ (colored in blue) predicted by PVQD and of the TM-scores_experiment_ (colored in salmon). The proteins are grouped according to the experimental methods used for determining their structures. (c) The distributions of the Pearson correlation coefficients between the residue-wise RMSFs in predicted conformational ensembles and the residue-wise displacements of backbone atom positions in the alternative experimental structures. The results for PVQD (blue), EigenFold (green), NeuralPLexer (pink) and MSA subsampling (salmon) are shown. The proteins were grouped according to their manually assigned types of conformational change. (d) Comparisons between PVQD, EigenFold, NeuralPLexer and MSA subsampling. The results listed are the mean/median values over the 90 proteins in apo-holo benchmark set and over the 77 proteins in the fold-switching set. (e) The distributions of the TM-scores_ensemble_ (obtained with the PVQD (blue), EigenFold (green), NeuralPLexer (pink) and MSA subsampling (salmon)) were compared with the distributions of TM-scores_experiment_ (gray). The proteins are grouped by their types of fold switching changes. The boxplots show median, interquartile range, and minimum and maximum values excluding outliers (>1.5 times the interquartile range beyond the box).

For proteins in the apo-holo set, the two highest TM-scores from predictions (averaged as TM-score_ensemble_ following Jing *et al*. [20]) were compared against TM-scores between experimental structures (TM-score_experiment_) in Fig. 4a. For 72 proteins, TM-score_ensemble_ exceeded TM-score_experiment_. Figure 4b compares distributions of TM-score_ensemble_ and TM-score_experiment_ with proteins grouped by a structure determination method (X-ray versus NMR/CryoEM groups). Similarly, Fig. S5A shows comparisons with proteins grouped by conformational change type. Across all groups, TM-score_ensemble_ distributes higher than TM-score_experiment_, indicating that approximation errors of PVQD-predicted structures are smaller than experimental conformational variations, thus PVQD recapitulated changes between alternative experimental structures for many benchmark proteins.

To semi-quantitatively examine recapitulation of structural variation, we computed per-protein Pearson's correlation coefficients between residue-wise RMSFs in PVQD-predicted structure ensembles and residue-wise backbone deviations between alternative experimental structures. Figure 4c shows the distribution of correlation coefficients grouped by conformational change type (determined via manual inspection): localized (‘local’), inter-domain motions and others. For all three groups, there are substantial fractions of proteins for which the PVQD ensemble recapitulated the experimental structural variations with relatively high Pearson's correlation coefficients.

We compared PVQD against three approaches: MSA subsampling (applying AlphaFold2 to MSA subsamples [13]), EigenFold (employing harmonic diffusion [20]) and NeuralPLexer (using multiscale generative modeling with sequence/structure templates [32]). For TM-score_ensemble_, PVQD (0.89) performs comparably to MSA subsampling (0.90) but outperforms EigenFold and NeuralPLexer (Fig. 4d). Regarding Pearson's correlations between ensemble RMSFs and experimentally observed structural changes, PVQD surpasses all methods (Fig. 4c and 4d). Protein-wise comparisons show PVQD outperformed EigenFold, NeuralPLexer and MSA subsampling in 64% (58/90), 67% (60/90) and 76% (68/90) of cases, respectively (Fig. S5B). Moreover, PVQD is single-sequence-based and is not limited by available MSA information.

For metamorphic proteins in the fold-switching set, PVQD showed lower average TM-score_ensemble_ values and RMSF-versus-displacement Pearson's correlations compared to the apo-holo set (Figs. 4d, 4e and S5C). Nonetheless, PVQD achieved TM-score_ensemble_ above TM-score_experiment_ values for 72% (60/77) of these proteins. Fold-switching changes were categorized as ‘local’ (TM-score_experiment_ above 0.7) or ‘global’ (TM-score_experiment_ below 0.7). For local changes, PVQD and MSA subsampling showed comparable TM-score_ensemble_ (average value of 0.73, median value of 0.78), outperforming other methods (Fig. 4d), with complementary results on some targets (Fig. S5D). PVQD exhibited higher displacement correlations (median Pearson’s coefficient value of 0.53) than subsampling (median Pearson’s coefficient value of 0.40) (Fig. S6C). For global changes, PVQD performance was suboptimal (median TM-score_ensemble_ of 0.61). Analysis of the global-change protein KaiB (see the next section) revealed PVQD may sample incomplete conformational states from a single sequence but can capture alternative conformation after mutations are introduced.

### Comparisons between predicted and experimental conformation sets with diverse structural dynamics

To further evaluate whether PVQD can recapitulate conformational landscapes for proteins with diverse structural dynamics, we selected four protein systems considering their biological significance and the representativity of their conformational dynamics, including: (1) K-Ras: a small GTPase molecular switch with frequent cancer-linked mutations inducing functionally relevant conformational changes [33–36,37]; (2) KaiB: the cyanobacterial circadian clock protein exhibiting metamorphic folding between a unique βαββααβ state and a thioredoxin-like βαβαββα state [38–40]; (3) 4.1G-CTD: an intrinsically disordered protein region interacting with disordered partners (e.g. NuMA during mitosis) [41,42]; and (4) D-allose binding protein: a multi-domain protein showing large ligand-induced transition, as indicated by E42 to D176 Cα distance from 17.7 Å in apo state (PDB: 1GUDA) shortened to 7.4 Å in holo state (PDB: 1RPJA) [43].

**Figure 5.**
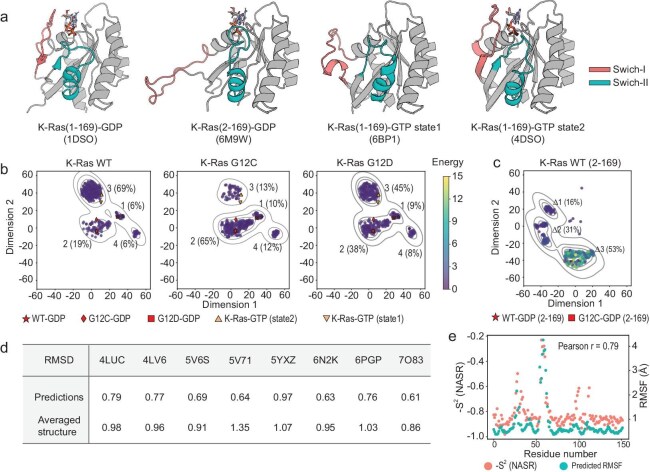
The conformational distributions of K-Ras predicted by PVQD. (a) Four representative experimental structures in distinctive conformational states with corresponding PDB IDs indicated. Two main variable regions of switch-I and of switch-II are colored in salmon and green, respectively. (b) Projections of the predicted conformations for different K-Ras variants into a 2D plane obtained by using t-SNE analysis of all predicted conformations. For clarity, the conformations predicted for different sequences are shown in different plots from left to right. The projected locations of 10 experimental structures are indicated using symbols below the plots. The predicted conformations shown in filled circles were colored according to their ABACUS2 energies (from yellow to purple). The clusters in the 2D plane are numbered from 1 to 4 and the percentage of conformations that fall into each cluster are indicated in parentheses. (c) The same as (b), but for predictions on the M1Δ variant. Projected locations of several experimental structures of M1Δ are shown with symbols as indicated below the plot. (d) The values of the lowest predictions-versus-experimental RMSD between 400 predicted conformations on the sequence of G12C mutant and 8 inhibitor-bound experimental structures of the G12C mutant. (e) The NMR-determined order parameters for WT K-Ras (colored in salmon) compared with the predicted RMSF computed on PVQD predicted structures (colored in green). The value of the Pearson correlation coefficient is indicated.

The conformational dynamics of the K-Ras GTP-binding protein are well-studied [33–37]. It cycles between inactive GDP-bound and active GTP-bound states during signaling [33], with structural differences concentrated in the switch-I/II regions. NMR revealed GTP-bound K-Ras exists in equilibrium between State 1 (inactive; promotes nucleotide exchange) and State 2 (active; facilitates GTP hydrolysis) (Fig. 5a) [33]. The switch-I/II conformations are modulated by ligands, mutations (e.g. cancer hotspot mutants G12C/G12D [35] with altered dynamics [37,44] or M1Δ variant lacking N-terminal Met with destabilized hydrophobic packing [36]) or post-translational modifications [33,36].

To evaluate the physical/functional relevance of PVQD-predicted conformations based on experimentally observed structures of K-Ras, we applied PVQD to generate 400 conformations per sequence for WT K-Ras and mutants G12C, G12D and M1Δ. Projection by t-SNE showed similarly located conformation clusters for full-length sequences, with variant-dependent population variations (Fig. 5b). Experimental structures were positioned as follows: WT GDP-bound structures (PDBs: 6MBU and 5W22) in cluster 1 (lowest prediction-experiment RMSDs: 0.69 Å and 0.84 Å, respectively); GTP-bound structures fell in cluster 3: inactive state structures (PDBs: 6ASE, 6BP1; lowest RMSDs: 1.93 Å, 1.94 Å) and active state structure 4DSO (lowest RMSD: 0.71 Å). The G12C GDP-bound structures (PDBs: 4LUC, 5V6S and 7O83) fell in cluster 2 (RMSDs: 0.61–0.79 Å), while the G12D GDP-bound structures (PDBs: 4EPR and 7EW9) were split between clusters 1 and 2 (lowest RMSDs: 0.60 Å and 0.73 Å, respectively). As predicted by PVQD, the population fraction in cluster 2 was mutation-dependent: highest for G12C (65%), lowest for WT (19%), and intermediate for G12D (38%). Thus, the PVQD-predicted clusters 1–3 recapitulated the function- or mutation-relevant K-Ras conformations from experimentally determined structures.

No experimental counterparts were identified for cluster 4 conformations sampled by PVQD. Fig. S6A shows the primary difference of these structures from WT GDP-bound structures lies in switch-II, with inhibitor binding pockets near switch-II being more compact (see Fig. S6B). These conformations also exhibit lower ABACUS2 energies than cluster 1 (1.50 versus 1.72) [45], suggesting they may represent physically plausible, though experimentally unobserved states.

Previous experiments have revealed that M1Δ variant's N-terminal methionine removal causes major conformational changes in switch-I [36]. Figure 5c shows PVQD-predicted M1Δ conformations differ from full-length distributions. Clusters Δ1 and Δ2 correspond to GEF-bound WT H-Ras structure 1BKD (RMSDs: 1.49 Å, 2.00 Å) [37], while cluster Δ3, absent in full-length predictions, matches experimental M1Δ structures 6M9W and 7F0W (lowest RMSDs: 1.00 Å, 1.04 Å). Thus, PVQD recapitulated the experimental impact of N-terminal truncation on K-Ras conformation.

To compare PVQD's predictions against ligand-bound states, we analyzed lowest RMSDs for eight experimental K-Ras G12C structures bound to various small molecules. Figure 5d shows every experimental structure was recovered with RMSD below 1.0 Å by at least one prediction. These lowest RMSDs were substantially smaller than RMSDs from the average experimental structure, indicating PVQD-sampled conformations could serve as alternative receptor structures for identifying state-specific binding pockets.

We compared experimental NMR S^2^ order parameters [37] with residue-wise RMSFs of PVQD-generated conformation sets for WT, G12C and G12D K-Ras to further assess the physical relevance of the sampled conformation distributions. The results (Fig. 5e and Fig. S7C) show high correlation (Pearson's coefficients above 0.76), further demonstrating the physical relevance of PVQD-predicted conformational fluctuations.

**Figure 6.**
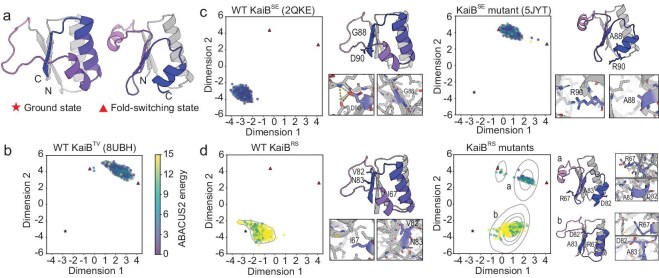
The conformational distribution of KaiB predicted by PVQD. (a) Two experimentally determined conformational states of KaiB. The region exhibiting main conformational change is highlighted with different colors. (b–d) Projections of the PVQD predicted conformations for three wild type KaiB homologs and two mutants. The conformations predicted for each sequence and three experimental structures were projected into the same 2D planes with t-SNE. The red stars and red triangles, respectively, correspond to structures in the ground state and in the fold-switched state. The predictions shown in filled circles were colored according to their ABACUS2 sequence energies (from yellow to purple). In (c) and (d) comparing the results for wild type and mutated sequences, representatives of conformational clusters in each 2D plot were shown next to the corresponding 2D plot with enlarged views of the structural details near the mutated residues.

As the next example, we considered KaiB, a cyanobacterial circadian oscillator subunit [38] that metamorphically transitions from a ground state fold to a fold-switched (FS) state upon binding with the protein KaiC [39,40]. The two states exhibit a TM-score of 0.49, indicating large conformational differences (Fig. 6a). Experimentally, the ground state structure was crystallized in isolation, while FS structures were determined in KaiB-KaiC complexes [39] or via specific mutants [40]. Mutants stabilizing FS states relative to ground states have been proposed, e.g. D90R and G88A in KaiB^SE^ homologs [46]. PVQD was applied to three KaiB homologs with known conformational states (KaiB^TV^[38], KaiB^SE^ [46] and KaiB^RS^ [47]). For each of five sequences, including the three wild types and two mutants (KaiB^SE^ D90R/G88A [39]; KaiB^RS^ V82D/N83A/I67R [48]), 400 conformations were predicted. For wild type, the predictions covered the ground and FS states (Fig. 6): KaiB^TV^ exclusively sampled the FS state (lowest RMSD: 0.72 Å to FS structure 8UBH; Fig. 6b), while KaiB^SE^ and KaiB^RS^ covered only the ground state (lowest RMSDs: 0.92 Å and 1.34 Å to ground structure 2QKE; Fig. 6c and 6d). For the KaiB^SE^ mutant, the predictions shifted to the FS state (Fig. 6c), consistent with mutant stabilization [39]. Predictions on the KaiB^RS^ mutant yielded two clusters: cluster a corresponded to the FS state (RMSD: 1.84 Å to FS structure 5JYT); cluster b represented a novel state with high ABACUS2 energies. Inspection of structures in the latter cluster revealed buried charged residue I67R in hydrophobic regions, suggesting physical instability.

PVQD was tested for predicting intrinsically disordered proteins using 4.1G57 CTD and a 4.1G57-NuMA16 fusion peptide (previous research has shown this peptide can form ordered but dynamic structures [49]). Ensembles (400 structures for each protein) were predicted and analyzed. The t-SNE projections in Fig. S7 shows two clusters: one disordered and one resembling the structured 4.1G57-NuMA16 conformation (PDB: 8I8Y; averaged RMSD 3.2 Å) containing one helix and three β-strands. In the RMSF plots, the fusion peptide exhibited reduced C-terminal conformational fluctuations versus isolated 4.1G57 CTD (RMSF from 6.98 Å to 2.81 Å; Fig. S7C), in agreement with the experimentally observed ordered but dynamic nature of the system [49].

For the D-allose binding protein, PVQD sampled open (apo-like) and closed (holo-like) states, with experimental structures falling within the sampled clusters (TM-scores: 0.96–0.97, Fig. S8A and S8B). Differences between the two clusters primarily corresponded to inter-domain motions, with small intra-domain fluctuations (Fig. S8C). Strong correlations between residue-wise RMSFs and domain-superimposed structural deviations (Fig. S8C and S8D) substantiated PVQD's recapitulation of intra-domain conformational fluctuations.

## DISCUSSION

The PVQD auto-encoder employs a latent space vector quantization approach originally developed for images and speech [16,22–24], compressing continuous high-dimensional data with high-frequency details into discrete tokens (structural codes) in a latent space. This achieves significant dimension reduction and distribution smoothening, facilitating efficient generative model learning for downstream applications. Sampling in this low-dimensional manifold enables high diversity data generation [16,21,22,24,25], while a well-trained decoder recovers missing fine details to complete the end-to-end model.

While the auto-encoder performs well for shorter proteins (below 500 residues), structural feature loss occurs in longer proteins (above 500 residues). Fig. S9 indicates that the increased total RMSDs come mainly from distortions at very large inter-residue distances (detailed in Supplementary Notes). This suggests the current graph-based encoder, emphasizing local residue interactions, only coarsely models long-range spatial relationships for extended conformations. Increasing the size of the codebook marginally improves large-protein recRMSD, but architectural modifications (e.g. multiscale/hierarchical graphs with global attention layers) may better encode long-range arrangements.

PVQD's discretized 1D encoding of 3D structures could serve various purposes (e.g. tokens in structural language models). Here we have only considered structure sampling via DDPM in the resulting latent space. As intended, PVQD overcomes key biases in direct–3D-diffusion methods [6,7,9], generating structures with higher β-strand composition, longer loops and more reasonably sized domains. These computationally designable structures are inherently less rigid than previous designs and can host richer conformational dynamics. This likely stems from smoother latent space distributions enabling easier unbiased DDPM training. Since conformational dynamics are essential for functions like catalysis and allostery, it will be of great interest to verify that such PVQD designed structures and their conformational dynamics can indeed be realized in future wet experiments.

PVQD's lightweight latent space DDPM enables efficient tuning for structure prediction and conformation sampling conditioned on a single sequence, thus unifying structure prediction and design. PVQD matches state-of-the-art single-sequence-based structure prediction in accuracy while uniquely enabling prediction of conformational distributions, which are valuable for studying functional dynamics. Compared with MSA perturbation approaches [11,13], DDPM-based sampling offers a single-sequence-based solution. For apo-holo benchmark proteins, PVQD approximated alternative experimental structures with errors smaller than inter-experimental differences, with 68/90 cases showing higher residue-wise RMSF correlations than MSA subsampling (median Pearson's coefficient: 0.59 versus 0.37). For fold-switching proteins, while some limitations of PVQD in predicting ensembles for global fold-switching proteins have been observed (detailed in Supplementary Notes), PVQD still performed comparably to state-of-the-art methods, especially for local changes. Examined systems (K-Ras, KaiB, 4.1G CTD, D-allose binding protein) confirmed PVQD-sampled conformations recapitulate functionally relevant states. Mutational effects on conformation distributions were also captured to some extent.

We note PVQD, trained without molecular dynamics (MD) simulation data, offers computational efficiency advantages over MD simulations. Unlike MD requiring initial structures and computationally intensive simulations, PVQD generates 10 distinct conformations for a 288-residue protein (PDB: 1RPJ) with RMSF 2.67 Å in 67 seconds using one NVIDIA 3090 GPU, contrasting MD's days-long simulations on a CPU cluster (Table S2). This provides a scalable, sequence-conditioned framework for rapid exploration of large structural landscapes or multi-state protein design, complementing MD. PVQD enables efficient exploration of natural protein conformational dynamics for functional insights and design of functionally flexible backbones. Our results establish PVQD's latent space diffusion as a comprehensive framework unifying protein structure prediction and design, offering significant enhancement in modeling conformational dynamics over deterministic prediction networks or direct 3D-space diffusion design approaches.

Comparisons with experimental structures of K-Ras, KaiB, 4.1G CTD and D-allose binding proteins indicate most PVQD-sampled conformations correspond to physically plausible states. However, some states lack experimental counterparts, and PVQD cannot inherently distinguish physically existing states from potential model artifacts. Evaluating PVQD predictions with computational tools (e.g. force fields or statistical energy functions) may resolve this. In this work, we found ABACUS2 energy-based selection of the top 50% of conformations effectively balances computational efficiency, physical plausibility filtering and conformational diversity preservation (Fig. S10; see Supplementary Methods and Supplementary Notes for details). Further developments of such integrated approaches may combine the computational efficiency and sampling power of deep generative models and the physical interpretability of conventional energy functions. Such progress will substantially enhance our ability to model the conformational dynamics of proteins.

## MATERIALS AND METHODS

### The auto-encoder of PVQD

PVQD's auto-encoder employs a graph-based encoder processing central residues and their 30 nearest neighbors. Node features include directional vectors; edge features encode sequence separation, Cα-Cα distance, direction and orientation. After transforming, the encoder outputs a 16-D latent vector per residue.

Latent vectors undergo vector quantization: a codebook of K vectors ${e}_k$ maps any vector $v\ $to its closest code ${e}_k$, namely,$\ \textit{Quantized}( v ) = {e}_k,{\mathrm{\ where\ }}k = {\mathrm{argmi}}{{\mathrm{n}}}_j\|v - {e}_{\mathrm{j}}{\|}_2$. This converts backbone ${\boldsymbol{x}}$ to code array ${q}_i\in 1{...} K$, with latent vectors ${\boldsymbol{e}} = E( {\boldsymbol{x}} )$. Then, the decoder reconstructs ${\boldsymbol{x}}$ from ${\boldsymbol{e}}$ with SE(3)-invariant blocks [2,8]. The parameters of the encoder network $E( \cdot )$, the codebook ${\boldsymbol{e}}$ and the decoder network $D( \cdot )$ are learned by optimizing reconstruction losses and vector quantization losses on the training backbones. For a backbone ${\boldsymbol{x}}$, the total loss is $L( {\boldsymbol{x}} ) = {L}_D( {{\boldsymbol{x}},D( {\boldsymbol{e}} )} ) + \|sg( {E( {\boldsymbol{x}} )} ) - {\boldsymbol{e}}\|_2^2 + \beta \|sg( {\boldsymbol{e}} ) - E( {\boldsymbol{x}} )\|_2^2$, in which the first term ${L}_D( {x,D( e )} )$ represents structure reconstruction loss (see Supplementary Methods), and the second and third terms represent vector quantization losses [23,25,26]. With the operator $sg$ referring to the stop-gradient operation that blocks the back propagation of error gradients, the second term leads to optimization of the codebook ${\boldsymbol{e}}$, while the third term leads to optimization of the encoder. The hyperparameter $\beta {\mathrm{\ }}$(chosen to be 0.25 in this work) controls the relative weights.

Training of the auto-encoder used above 4.0 Å-resolution PDB monomer structures (until April 30, 2020), clustered at 30% sequence identity, split into training (22 378 clusters), validation (1525 clusters) and test (1451 clusters) sets. The training contains two phases. Initial training updates parameters for 100k steps using contiguous 256-residue segments; fine-tuning only the decoder for an additional 100k steps using 640-residue segments.

### The diffusion-based generator of PVQD

PVQD models latent space distributions of natural backbones using a DDPM with a DiT-based denoising network [50]. The diffusion module generates latent vectors from Gaussian noise, which are decoded into 3D structures. Training minimized MSE between generated and corresponding native latent vectors using the pre-trained auto-encoder.

For sequence-conditioned structure prediction, the unconditional DDPM was fine-tuned by incorporating amino acid sequences via encoding sequences with ESM (650M parameters) and introducing multi-head cross-attention after self-attention in each DiT block (Fig. S11). We fine-tuned DDPM for 100k steps while excluding training samples with >70% sequence identity to all benchmark targets.

Detailed protocols used for structure generation, conformational evaluation and energy analysis are provided in Supplementary Methods.

## Supplementary Material

nwaf290_Supplemental_File

## CODE AVAILABILITY

The scripts and all prediction results are available on https://github.com/liuyf020419/PVQD-torch.git. The codes are available in two versions: one based on the PyTorch (https://github.com/liuyf020419/PVQD-torch.git) framework and the other based on the MindSpore (https://github.com/liuyf020419/PVQD-mindspore.git) framework.
