# Supplementary material for: Designing flexible protein structures and sampling protein conformations with a unified model using vector quantization and diffusion
Source: Natl Sci Rev. 2025 Jul 16;12(11):nwaf290. doi: 10.1093/nsr/nwaf290 (PMC12618002; doi:10.1093/nsr/nwaf290)
Supplement: nwaf290_Supplemental_File [file nwaf290_supplemental_file.docx]

Supplementary Materials for

**Designing Flexible Protein Structures and Sampling Protein Conformations with a Unified Model using Vector Quantization and Diffusion**

Yufeng Liu^1,2^, Linghui Chen^3^, Quan Chen^1,2^, Haiyan Liu^1,2,4,5*^

^1^MOE Key Laboratory for Membraneless Organelles and Cellular Dynamics, School of Life Sciences, Division of Life Sciences and Medicine, Hefei National Research Center for Physical Sciences at the Microscale, University of Science and Technology of China, Hefei, Anhui, 230001, China.

^2^Anhui Basic Discipline Research Center of Artificial Intelligence Biotechnology and Synthetic Biology, University of Science and Technology of China, Hefei, Anhui 230027, China.

^3^Oristruct Biotech Co., Ltd, Hefei, Anhui 230026, China

^4^School of Biomedical Engineering, Suzhou Institute for Advanced Research, University of Science and Technology of China, Suzhou, Jiangsu 215127, China

^5^Department of Ocean Big Data and Prediction, Laoshan Laboratory, Qingdao, China

*To whom correspondence should be addressed.

e-mail: hyliu@ustc.edu.cn

[Supplementary Methods 3](#_Toc201996621)

[Structure reconstruction loss for training the auto-encoder of PVQD 3](#_Toc201996622)

[Predicting conformational ensembles with different methods 4](#_Toc201996623)

[Energy evaluation on generated conformations from PVQD 5](#_Toc201996624)

[Supplementary Notes 5](#_Toc201996625)

[Ablation study for different configurations of DDPM 5](#_Toc201996626)

[Analysis of structural features causing reconstruction errors 6](#_Toc201996627)

[Analysis of PVQD’s sampled conformational diversity versus experimentally observed alternative conformations 7](#_Toc201996628)

[Energy analysis and filtering of conformations sampled by PVQD 7](#_Toc201996629)

[References 25](#_Toc201996630)

Supplementary Methods

Structure reconstruction loss for training the auto-encoder of PVQD

The total structure reconstruction loss is composed of the Frame Align Point Error (FAPE) loss as used by AlphaFold2 (*1*) supplemented with four auxiliary losses: a distogram classification loss ($CE_{dist}$), a backbone conformation loss ($MSE_{BBconf}$), a covalent geometry violation loss ($MSE_{violation}$) and an amino acid type recovery loss ($CE_{aatype}$). That is,

$L_{D}\left( \boldsymbol{x},D\left( \boldsymbol{e} \right) \right)=FAPE +CE_{dist} + MSE_{BBconf} + MSE_{violation} + CE_{aatype}$. (1)

Here, the amino acid types were predicted from the residue-wise representation in the last IPA block of the structure decoder, while the categorized inter-atomic distances were predicted from the pair representation of the same IPA block.

We note that while the squared error losses FAPE, $MSE_{BBconf}$ and $MSE_{violation}$ are based on the reconstructed three-dimensional structures, the cross-entropy losses $CE_{dist}$ and $CE_{aatype}$ are computed with separate classifiers decoding respectively inter-atomic distance categories and amino acid types based on the last internal layer of the structure decoder.

**Computational details about structure generation and evaluation**

With each of the five models compared for unconditional backbone generation, we performed 100 independent runs for each of the lengths of 70, 100, 200, and 400 residues. Program sources and computational details of using the various methods are described below.

1. RFdiffusion: We used the script and parameters provided in <https://github.com/RosettaCommons/RFdiffusion.git> with default settings.
2. Chroma: We locally installed the package provided in <https://github.com/generatebio/chroma.git> and used the API with default settings.
3. SCUBA-D: We used the source code and the scripts for unconditional generation provided in SCUBA-D (https://github.com/liuyf020419/SCUBA-D.git) that generate structures with 400 times steps.
4. PVQD: We used the learnt parameter of the generator of PVQD and followed the schedule that decreased the time step from 400 to 0 step by step.
5. PVQD+SCUBA-D: The initial backbone was generated with PVQD following the above settings in the first phase. Then the initial backbones were used as input to SCUBA-D to obtain refined backbones.

The TMalign program (*2*) was from https://zhanggroup.org/TM-align. The ESMFold program (*3*) from <https://github.com/facebookresearch/esm.git>. The ProteinMPNN program (*4*) was from <https://github.com/dauparas/ProteinMPNN.git>, and the program version was the one using 48 edges and 0.20 Å noise and with the default configuration for designing monomer. Foldseek program (*5*) was downloaded from <https://github.com/steineggerlab/foldseek.git> and used to search for similar structures in PDB and to determine the highest TM-score to PDB of generated backbones. The DomBpred program (*6*) was downloaded from <https://github.com/iobio-zjut/DomBpred.git> and used to assign the boundary of structure domains for the generated backbones.

Predicting conformational ensembles with different methods

Unless otherwise specified, we performed 40 independent runs with the corresponding programs for each target. For the DDPM in PVQD, we used a schedule that decreased the time step from 400 to 0 step by step. For the EigenFold, we used the scripts and default configurations provided in https://github.com/bjing2016/EigenFold. For the NeuralPLexer, we used the scripts and default configurations provided in https://github.com/zrqiao/NeuralPLexer. For MSA subsampling, AlphaFold2 was used to predict conformations with variably sampled subsets of the MSA by following the protocol and scripts provided in <https://github.com/delalamo/af2_conformations.git>. We controlled the depth of MSA through two parameters ‘*max_msa_clusters*’ and ‘*max_extra_msa*’ as defined in that protocol. The parameter ‘*max_msa_clusters*’ determining the number of randomly sampled sequence clusters used by AlphaFold2 for predicting one structure was set to 24. The parameter ‘*max_extra_msa*’ determining the number of extra sequences (referred to the as depth of the MSA) used for computing additional summary statistics was set to 48.

Energy evaluation on generated conformations from PVQD

The Rosetta energies were calculated using the RosettaDesign program v.3.12. All sidechains of all evaluated sequences (that is, the native sequences and the redesigned sequences) have been repacked by using the FixBB option of Rosetta, and then the complete structures have been relaxed using Rosetta RelaxBB to determine the energies.

The ABACUS2 energies were calculated using the ABACUS2 program (<http://biocomp.ustc.edu.cn/servers/abacus-design.php>). The sidechains of all evaluated sequences have been repacked by using ABACUS2 before calculating all-atom energies.

Supplementary Notes

Ablation study for different configurations of DDPM

We also performed ablation study to explore different configurations of DDPM, including noise schedules (linear, cosine, quadratic) and denoising step (200, 400, 800). We evaluated these configurations by training three distinct models with identical network architectures and the same 800 diffusion steps but varying the noise schedules. After updating parameters in the same steps, we applied three ablated models to respectively generate backbones of 70 to 200 residues with 200, 400 and 800 denoising steps. These generated backbones were quantified for structure quality using self-consistent TM-scores (scTM-scores) derived from ESMFold predictions and assessed for diversity through calculating mutual TM-scores between each other (We note that the possible value of a TM-score is between 0 to 1 with a higher value representing closer structure resemblance). The results (in Fig. S12) showed that the linear schedule outperformed cosine and quadratic variants in quality-diversity trade-offs, consistent with prior work that performs protein structure diffusion on 3D space (*7, 8*). Specifically, the linear noise schedule with 800 denoising steps achieved superior structure quality (averaged scTM-score of 0.75), maintaining high conformational diversity (averaged mutual TM-score of 0.32). Reducing the denoising step to 400 led to slightly decreased structure quality (averaged scTM-score of 0.74) but improved conformational diversity (averaged mutual TM-score of 0.29). To balance structure quality and conformational diversity, we finally selected linear schedule with 400 diffusion steps as DDPM configuration in PVQD.

Analysis of structural features causing reconstruction errors

To characterize this reconstruction error, we performed analyses across two categories of structural features: (1) local motifs (β-sheets, long loops, domain interfaces) and (2) global structural properties (structural domain count, structural domain size, CATH classification, and radius of gyration) (see Fig. S9). Residue-wise analysis using local Distance Difference Test (lDDT) scores revealed that while residues in longer proteins exhibited lower lDDT scores (0.89 versus 0.95 for shorter proteins), no significant reconstruction bias was observed for β-sheets, long loops, or domain interfaces (Figs. S9A to S9C). Global structural analysis further demonstrated similar performance across different CATH architectures (mainly Alpha: 1.05Å, mainly Beta: 0.87Å, Alpha Beta: 0.78Å, see Fig. S9D) but identified two key correlations: multi-domain proteins showed higher recRMSD than single-domain counterparts (0.68Å versus 2.14Å for shorter proteins; 1.80Å versus 2.51Å for longer proteins; while increased domain size alone did not significantly impact accuracy, see Figs. S9E and S9F), and radius of gyration (*Rg*) exhibited strong correlation with reconstruction error for proteins in different length (Pearson coefficients of 0.72 for shorter proteins; Pearson coefficients of 0.69 for longer proteins, Fig. S9G). For example, an extended multi-domain protein structure of 2XQHA (*Rg* of 55Å) has recRMSD of 1.89Å, while a single-domain structure from the same CATH architecture 3ULTA (*Rg* of 13Å) achieves recRMSD of 0.78Å (Fig. S9H). These results suggest that the current graph-based encoder, which emphasizes local interactions within spatial neighbor residues, inadequately models long-range spatial relationships critical for reconstructing extended conformations. While increasing the codebook size can marginally improve recRMSD for large proteins, architectural modifications, such as multiscale or hierarchical graph construction with both local and global attention layers, may better capture long-range arrangements.

Analysis of PVQD’s sampled conformational diversity versus experimentally observed alternative conformations

Analysis on Euclidean distances between experimentally observed alternative conformations and maximum inter-conformation distances among PVQD-generated structures showed that PVQD’s sampled conformational diversity (average maximum distance of 17.21) falls short of the experimentally observed divergence for fold-switching transitions (averaged inter-conformation distance in latent space of 21.4) by a large margin (Fig. S13). These findings suggest that improving PVQD’s performance on fold-switching systems will likely require fine-tuning the model with additional experimental fold-switching transitions data.

Energy analysis and filtering of conformations sampled by PVQD

We applied Rosetta energy to rank 40 predicted conformations for each sequence. However, we found selecting only the lowest-energy predictions (top 10% in energy) for analysis can led to significant inferior performance in TM-score_ensemble_ (Fig. S10), likely due to energy function is very sensitive to small local structural deviations that reduced conformational diversity (average mutual TM-scores increased from 0.91 for top 50% predictions to 0.94 for top 10%). Therefore, we adopted a balanced threshold retaining the top 50% conformations based on energy criteria that can balance between physical plausibility and structure diversity.

Parallel ABACUS2 energy analysis demonstrated consistent trends, with selected top 10% conformations showing substantial energy reductions (from 3.73 to 2.44 per residue for apo-holo proteins and from 8.86 to 5.73 for fold-switching proteins). Notably, ABACUS2 energies exhibited strong correlation with Rosetta energies (*e.g.*, Pearson’ s r of 0.60 for apo-holo proteins, see Fig. S10), while being computationally more efficient. For a 288-residue protein (PDB ID: 1RPJ), ABACUS2 calculations required only 25 seconds compared to around 4 minutes for Rosetta energy using 5 processors.


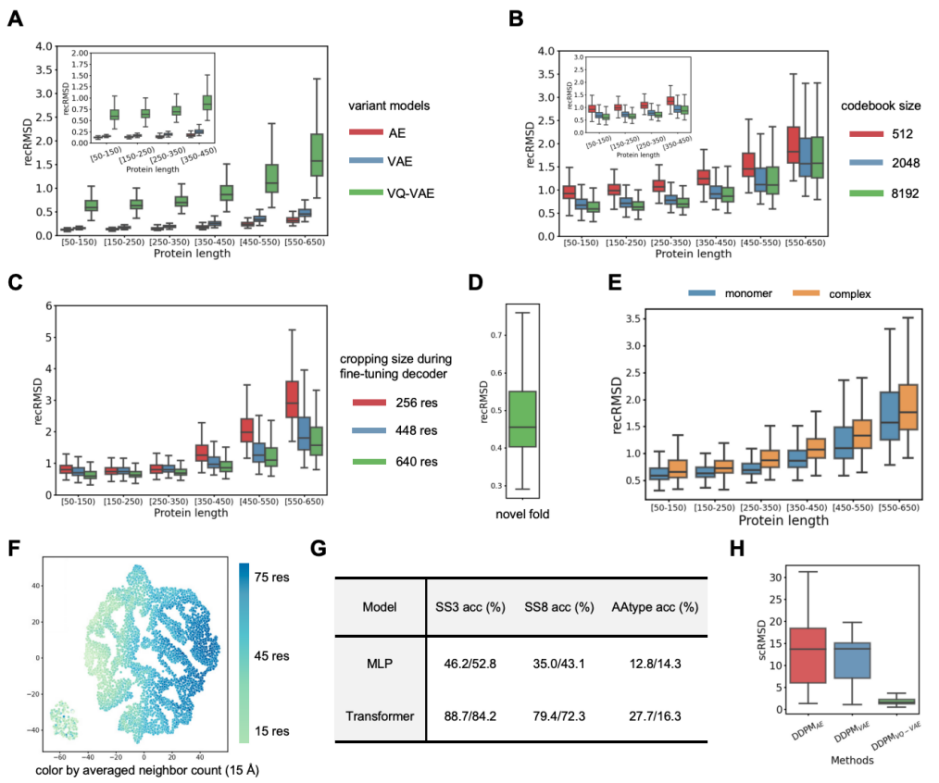


**Fig. S1. Performances and properties of the structure auto-encoder.**

**A.** The distributions of the RMSDs between natural and reconstructed backbones (recRMSDs) from three variant auto-encoder models. The results are for a set of non-redundant natural protein structures not used for training. **B.** The recRMSDs for the “VQ-VAE” model trained with different codebook sizes. **C.** The recRMSDs for the “VQ-VAE” model with the decoder fine-tuned with training samples of different crop sizes. **D.** The distributions of the recRMSDs for the set of non-natural backbones generated with SCUBA-D. **E.** Comparison of distributions of recRMSDs between monomer backbone and protein complex backbones for a set of non-redundant protein complexes from the PDB. **F.** Two-dimensional t-SNE projections of the quantization vectors contained in the codebook. Each point is colored according to the averaged counts of neighboring residues (15 Å radius) for residues assigned to the corresponding codes. We note that the small cluster at the lower left corner comprises of codes for residues in segments that lack through-pace contacts, *e.g.*, an isolated helix. **G.** Accuracies of two classifiers for classifying the residue-wise attributes from the array of the quantization vectors. One classifier is a multiple-layer perceptron (MLP) of three hidden layers that takes a single residue-wise vector as input. The other classifier is a Transformer of three repeated self-attention blocks that takes the array of vectors as input. Both classifiers were trained on the same training and validation set as used in training the auto-encoder up to the same number of 5k updating steps. The attributes include the secondary structure (in three states, SS3; and in eight states, SS8) and amino acid types (AAtype). The results were averaged over on training set/validation set. **H.** Distributions of scRMSDs of 100-residue backbones unconditionally generated with DDPMs in the latent space learnt by different auto-encoders. The boxplots show median, interquartile range, and minimum and maximum values excluding outliers (>1.5 times the interquartile range beyond the box).


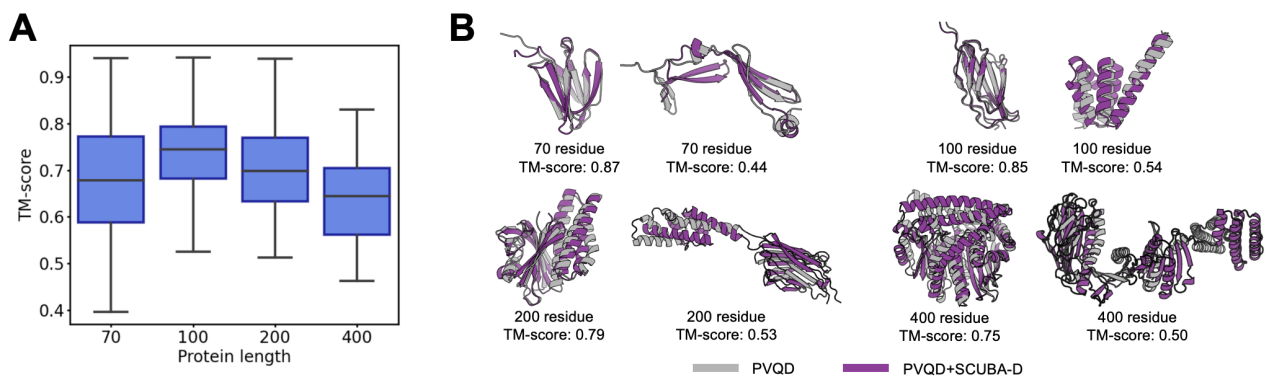


**Fig. S2. Results of using SCUBA-D to refine PVQD generated backbones.**

**A.** The distributions of TM-scores between the PVQD generated backbones and the corresponding backbones after refinement with SCUBA-D. The boxplots show median, interquartile range, and minimum and maximum values excluding outliers (>1.5 times the interquartile range beyond the box). **B.** Eight examples for pairs of PVQD generated backbones (colored in gray) and corresponding backbones after refinement (colored in purple). The value of TM-score for each pair are indicated.


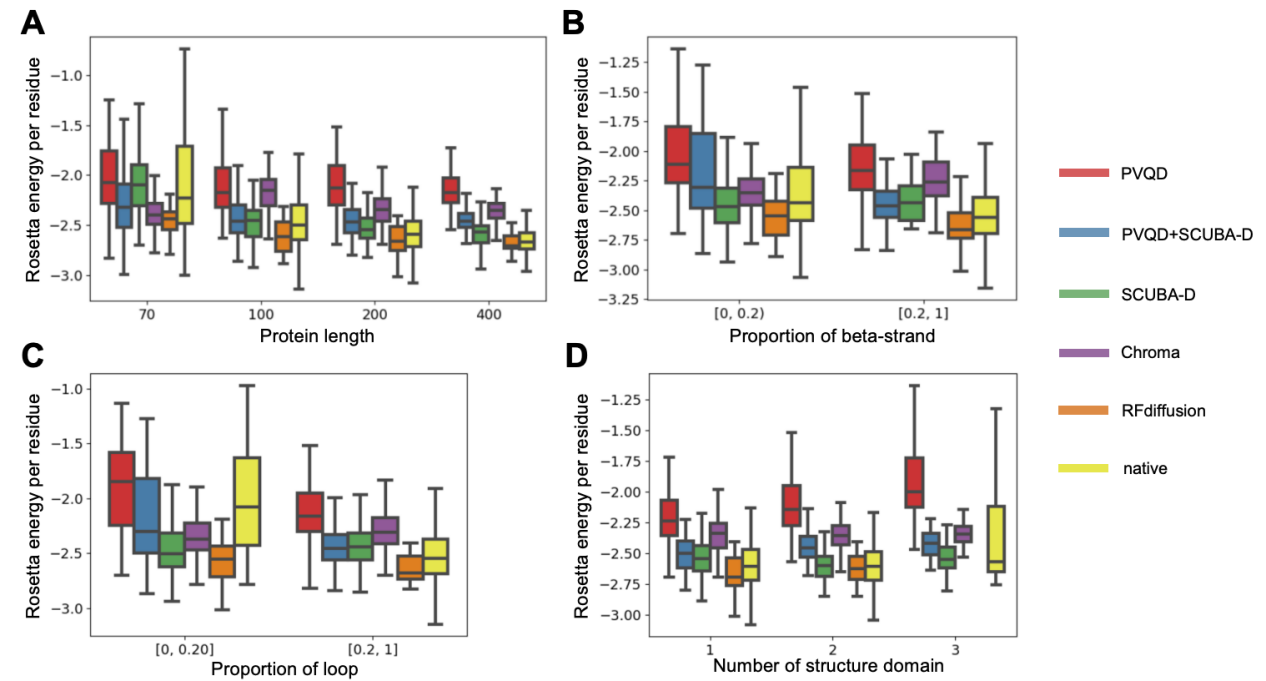


**Fig. S3. Energy evaluation on unconditional generated backbones from PVQD.**

**A.** The distributions of the Rosetta energy per residue for native backbones (yellow) and unconditionally generated backbones from PVQD (red), PVQD+SCUBA-D (blue), SCUBA-D (green), Chroma (purple) and RFdiffusion (orange). The backbones are grouped according to their length. **B.** The same as **A.**, but all backbones are grouped according to proportion of beta-strands. **C.** The same as **A.**, but all backbones are grouped according to proportion of loop segments. **D.** The same as **A.**, but all backbones are grouped according to the number of structure domains. The boxplots show median, interquartile range, and minimum and maximum values excluding outliers (>1.5 times the interquartile range beyond the box).


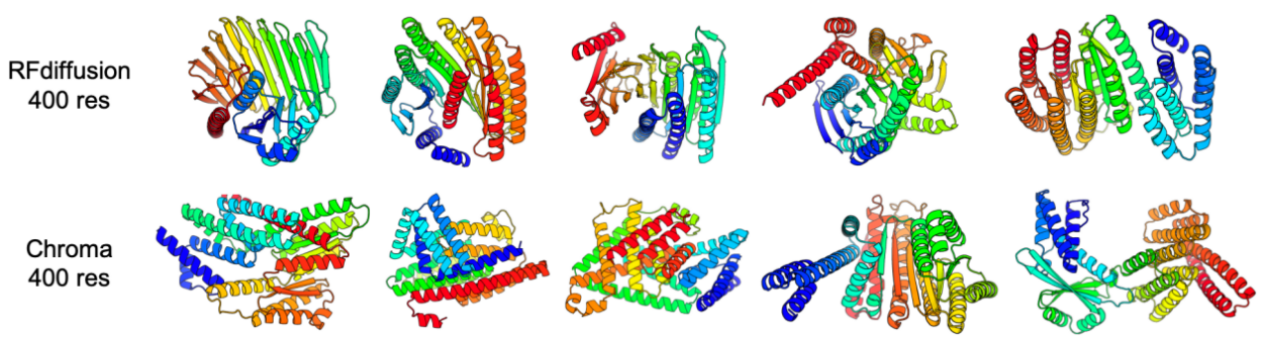


**Fig. S4. Examples of 400-residue backbones unconditionally generated with RFdiffusion and Chroma.**


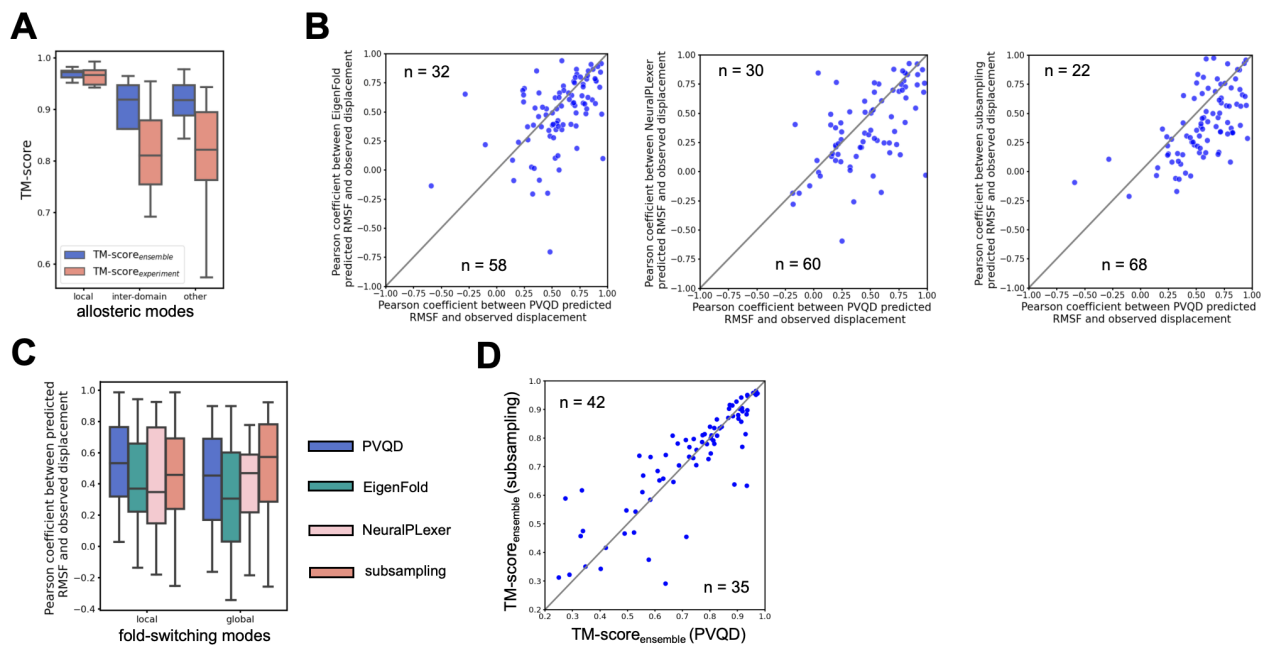


**Fig. S5. The performance of PVQD for the prediction of multiple multi-conformations for two benchmark sets of natural proteins.**

**A.** The distributions of the averaged highest TM-scores between the experimental and the PVQD-predicted structures (*i.e.,* TM-scores_ensemble_) compared with the distributions of TM-scores between the experimental structures (*i.e.,* TM-scores_experiment_). The proteins are grouped by their types of conformational changes. **B.** Protein-wise comparisons for PVQD and remaining approaches over 90 proteins in apo-holo benchmark set. From left to right: EigenFold, NeuralPLexer and MSA subsampling. The Pearson correlation coefficients are between the residue-wise RMSFs from the predicted ensembles and the displacements of atomic positions from the experimental structures. **C.** The distributions of the predicted-versus-observed Pearson correlation coefficients. The results for PVQD (blue), EigenFold (green), NeuralPLexer (pink) and MSA subsampling (salmon) are shown. The proteins were grouped into two categories according to their TM-scores_experiment_ above 0.7 or not. The boxplots show median, interquartile range, and minimum and maximum values excluding outliers (>1.5 times the interquartile range beyond the box). **D.** Protein-wise comparisons of the TM-scores_ensemble_ between PVQD and MSA subsampling approach over 77 metamorphic proteins in the fold-switching benchmark set. MSA subsampling approach outperforms PVQD for 42 proteins in the upper triangle, and PVQD outperforms MSA subsampling for 35 targets in the lower triangle.


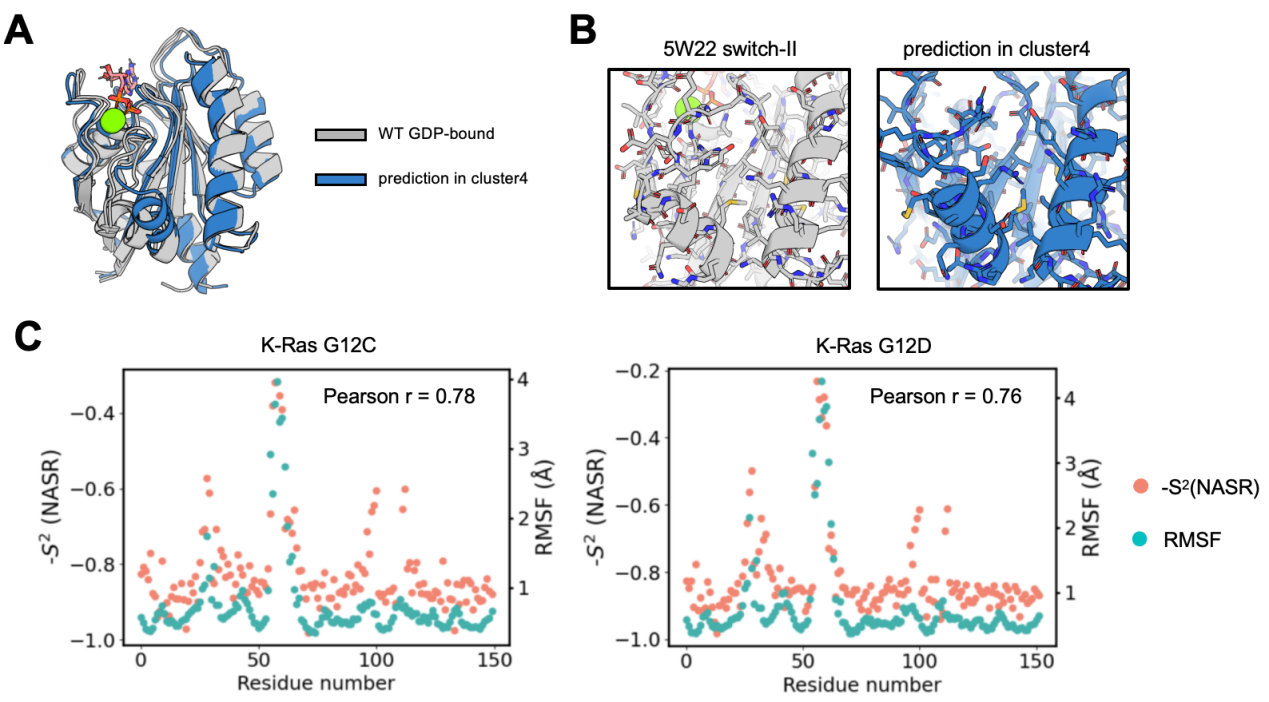


**Fig. S6. The representative PVQD-predicted conformation located in cluster 4 and comparison between NMR-determined order parameters and predicted RMSF obtained with PVQD on G12C and on G12D mutants.**

**A.** Two experimental structures of WT GDP-bound K-Ras (colored in gray, PDB ID: 5W22 and 6MBU) superimposed with a representative predicted conformation located in cluster 4 (colored in blue). **B.** Detailed views of the switch-II region for the WT GDP-bound K-Ras (5W22) and for the predicted conformation. **C.** The NMR-determined order parameters (colored in salmon) for two K-Ras mutants compared with corresponding residue-wise RMSFs computed on PVQD predicted structures (colored in green). The values of Pearson correlation coefficients are indicated.


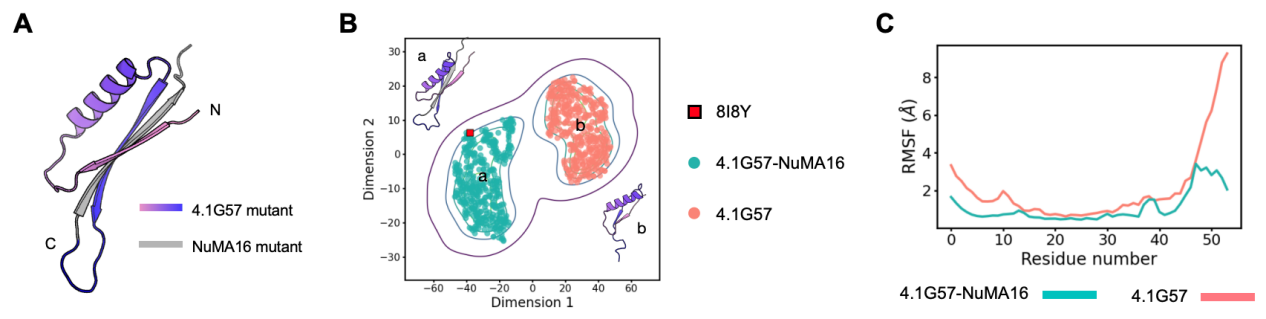


**Fig. S7. The conformational distributions of intrinsically disordered C-terminal domain (CTD) of 4.1G57 and 4.1G57-NuMA16 fusion protein predicted by PVQD.**

**A**. Experimental structures of the 4.1G57-NuMA16 fusion mutant (PDB ID: 8I8Y). The region from 4.1G57 CTD that exhibits main conformational change is highlighted with different colors. The region from NuMA16 is colored in gray. **B.** Projections of the PVQD predicted conformations for 4.1G57 CTD only (salmon) and 4.1G57-NuMA16 fusion protein (green). The conformations predicted for each sequence and one experimental structure were projected into the same 2D planes with t-SNE. The red square corresponds to experimental structures of the 4.1G57-NuMA16 fusion mutant. **C.** The residue-wise RMSFs of the predicted structures for 4.1G57 CTD only (salmon) and for 4.1G57-NuMA16 fusion protein (green).


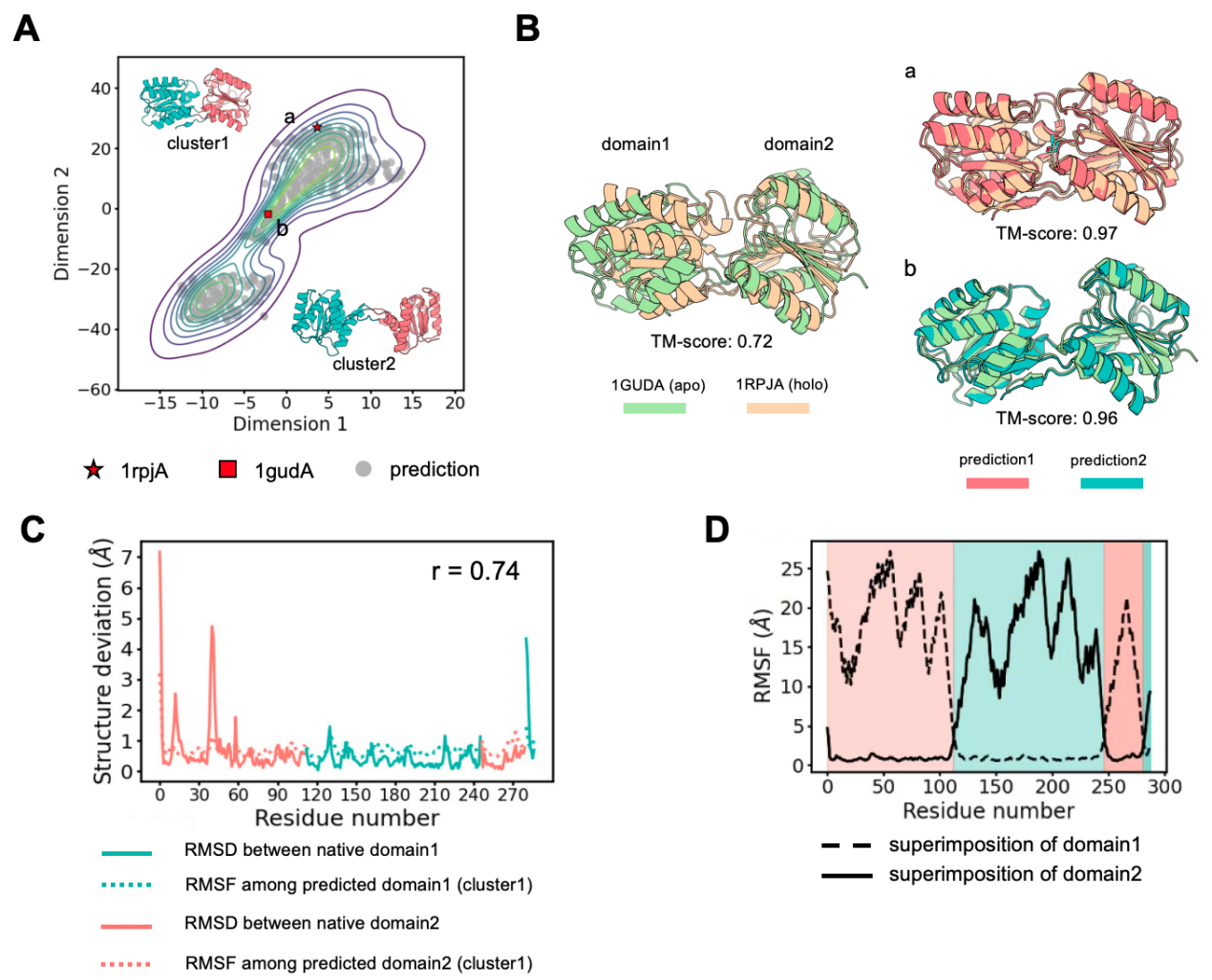


**Fig. S8. The conformational distributions of allose binding protein predicted by PVQD.**

**A.** Projections of 400 PVQD predicted conformations for allose binding protein. The conformations predicted for the sequence and two experimental structures were projected into the same 2D planes with t-SNE. The red square and red star respectively correspond to structures in apo state and structure in holo state. The representative structures of the two clusters are shown in two colors: domain 1 (residues 0-112 and 246-280, colored in green); and domain 2 (residues 112-246 and 280-287, colored in salmon). **B.** The superimpositions of the two labeled experimental structures for the D-allose binding protein (left) and of each experimental structure and its most similar (of the lowest RMSD) predicted structures (right up: 1RPJA; right down: 1GUDA). The TM-scores between the superimposed structures are indicated. **C.** The residue-wise RMSFs of the predicted structures in cluster 1 (dashed line) compared with the observed displacements of backbone atom positions in the experimental structures (solid line). The Pearson correlation coefficient is indicated. The RMSFs and displacements have been computed by superimposing the residues of domain 1 and domain 2 separately. The plots are colored in red for residues in domain 1, and in green for residues in domain 2. **D.** The residue-wise RMSFs of backbone atomic positions in the predicted structures. The solid (dashed) line was computed by superimposing the residues in domain 1 (2). The residue ranges spanned by domain 1 (2) were shadowed in salmon (green).


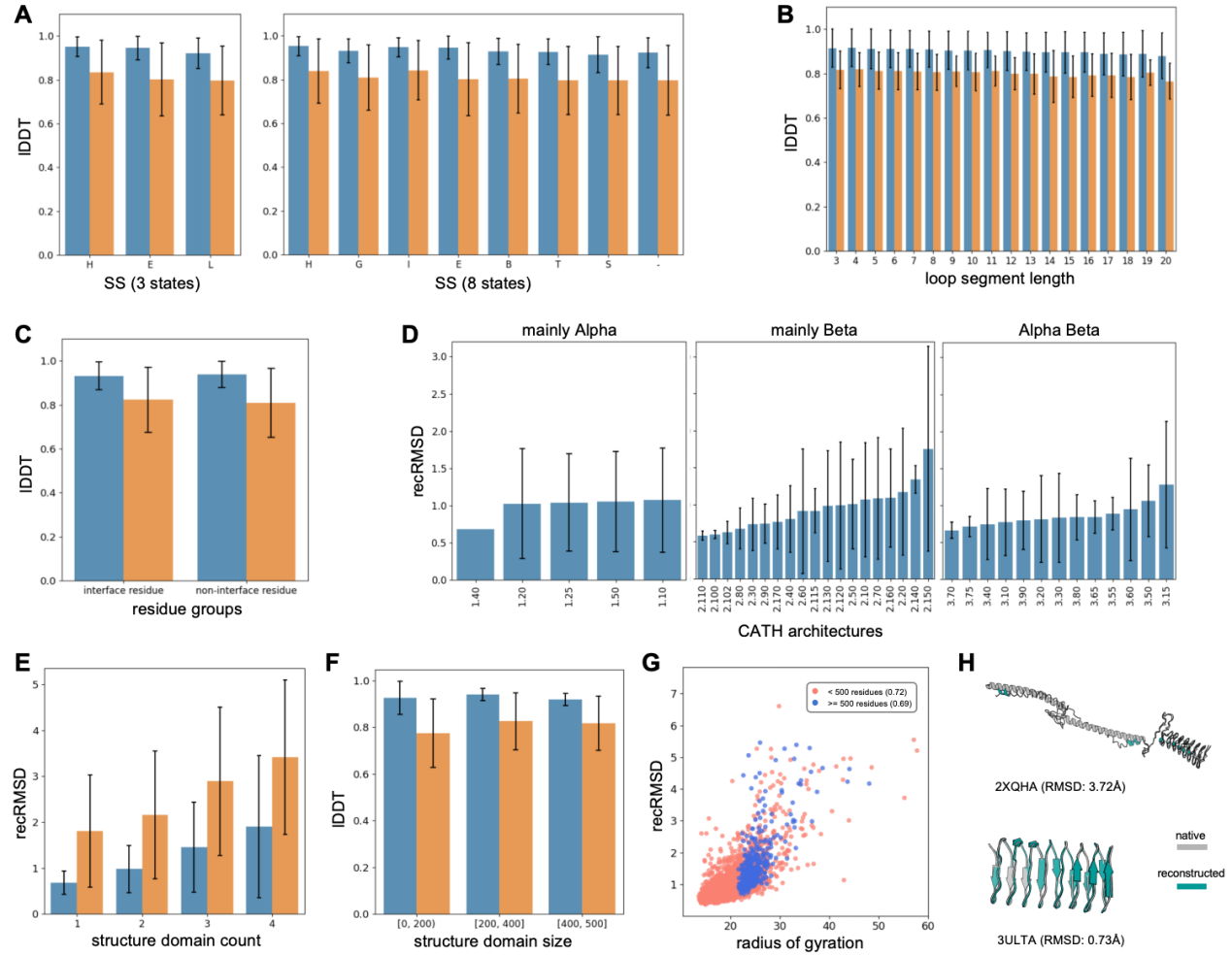


**Fig. S9. Effects of structural features on reconstruction accuracy of PVQD auto-encoder.**

**A.** The local Distance Difference Test (lDDT) scores for residues with secondary structure in three states (SS3) and in eight states (SS8) from shorter proteins of less 500 residues (blue) and from longer proteins of more than 500 residues (orange). **B.** The lDDT scores for residues in loop segments of different lengths. **C.** The lDDT scores for residues within or outside of structural interfaces. The interface residues refer to those spatially close to any residues (below 8.5Å) in other structural domains. **D.** The RMSDs between natural backbones and reconstructed backbones (recRMSDs) for proteins of distinct CATH architectures. **E.** The recRMSDs for proteins containing different counts of structure domains. **F.** The averaged lDDT scores for structure domains of different lengths. **G.** Scattering plot of recRMSD versus radius of gyration for proteins of shorter lengths (salmon) and of longer lengths (blue). The Spearman correlation coefficients are indicated. **H.** Superimpositions of two example protein structures in CATH architecture of 2.150 (respective PDB IDs 2XQHA and 3ULTA) (colored in gray) with the reconstructed structures (colored in green). For barplots from **A.** to **F.**, the bars represent mean values, and the error bars represent the standard deviations (SD).


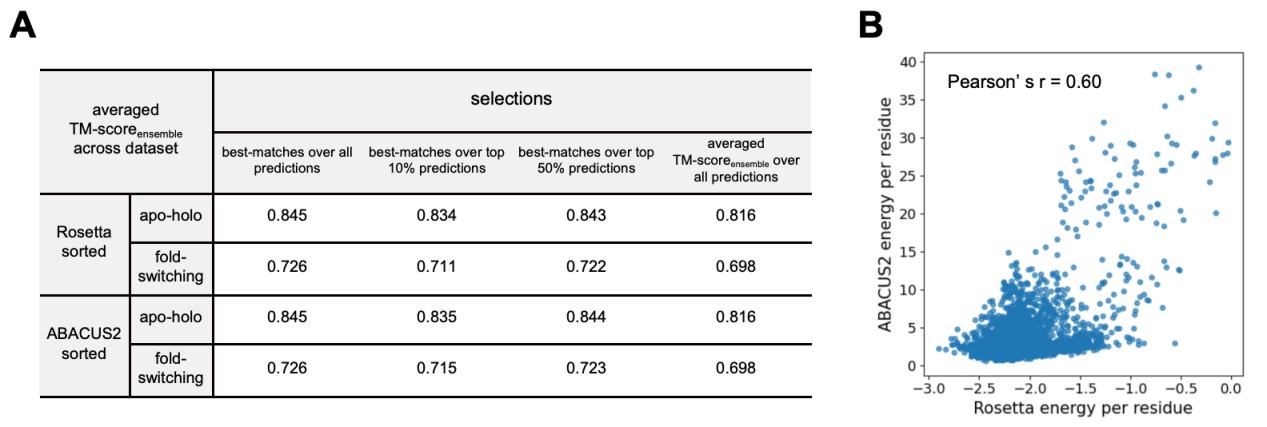


**Fig. S10. Energy evaluation on predicted conformations from PVQD.**

**A.**The averaged TM-score_ensemble_ for PVQD-predicted conformations across 90 proteins from the apo-holo dataset and 77 proteins from the fold-switching dataset. The values of TM-score_ensemble_ were calculated with different selection criteria over 40 predicted conformations. The selection criteria include: best-matches over all predictions, best-matches over top 10% predictions, best-matches over top 50% predictions and averaged TM-score_ensemble_ over all predictions. **B.** Scattering plot of ABACUS2 energy per residue versus Rosetta energy per residue for predictions on sequences from apo-holo benchmark dataset. The Spearman correlation coefficient is indicated.


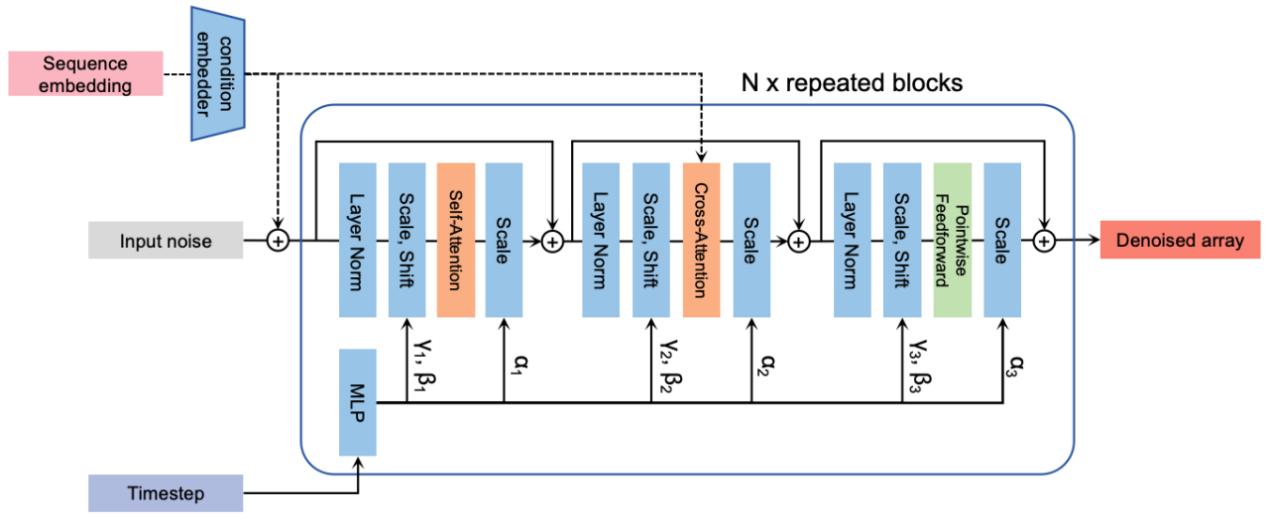


**Fig. S11. The architecture of the sequence-conditioned latent-space denoising module in PVQD.**


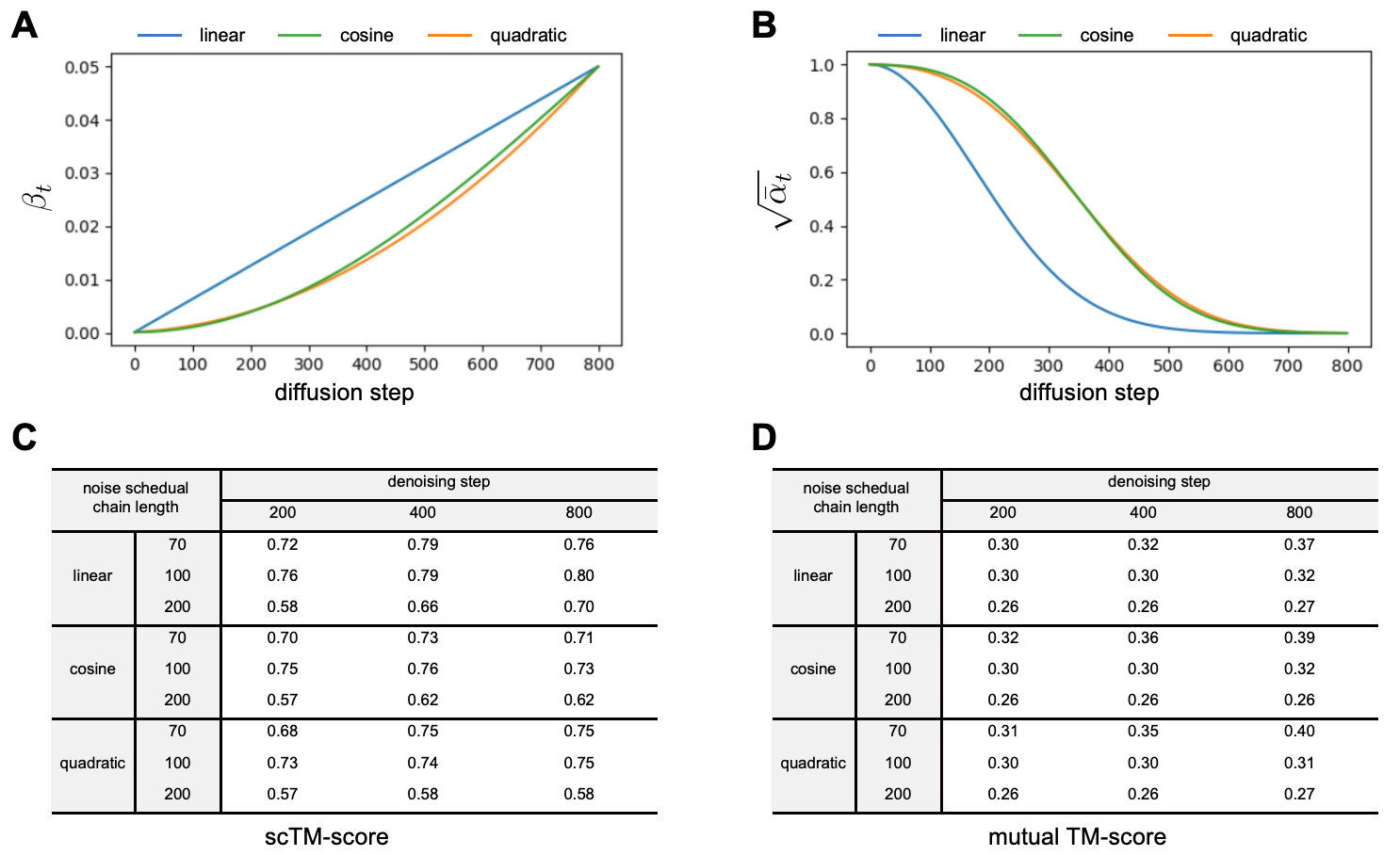


**Fig. S12. Comparisons of different configurations of DDPM in unconditional backbone generation.**

**A.** The trajectories of beta schedules in three DDPM configurations (blue: linear; green: cosine; orange: quadratic). **B.** the same as **A.**, but for trajectories of signal schedules in three DDPM configurations. **C.** The averaged scTM-score for 100 generated backbones of 70 to 200 residues sampled from different DDPM configurations. **D.** the same as **C.**, but for the averaged mutual TM-score.


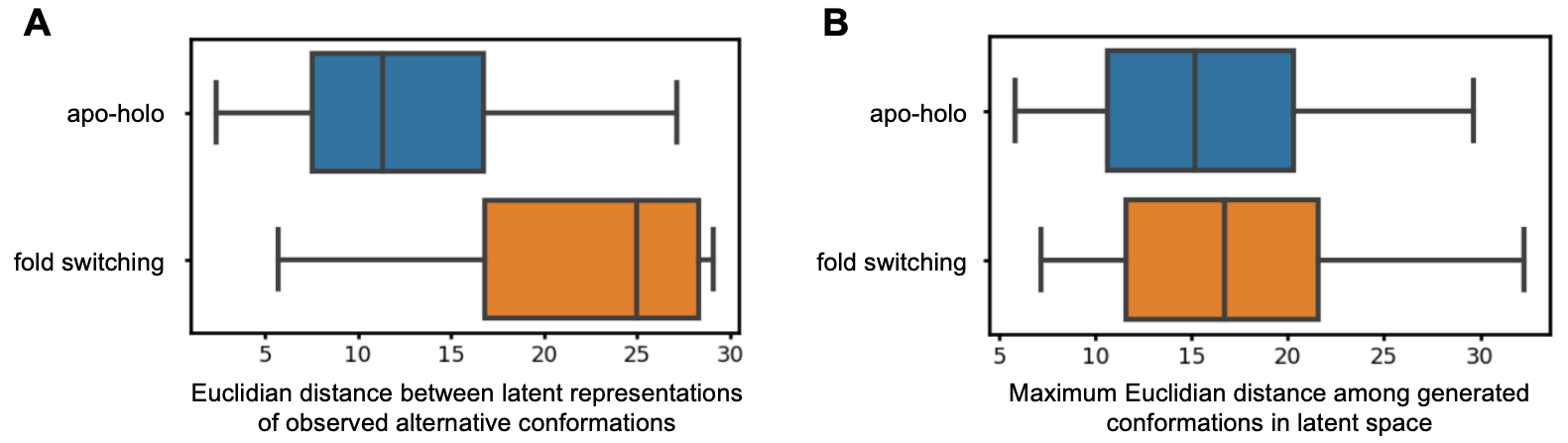


**Fig. S13. Comparisons between Euclidean distances of experimentally observed alternative conformations and of maximum inter-conformation distances among PVQD-generated structures in latent space.**

**A.** The distributions of the Euclidian distance between latent representation of observed alternative conformations for proteins in the apo-holo benchmark set (blue) and the fold-switching benchmark set (orange). **B.** The distributions of the maximum inter-conformation distance in latent space for 40 PVQD-generated structures per sequence in the apo-holo benchmark set (blue) and the fold-switching benchmark set (orange). The boxplots show median, interquartile range, and minimum and maximum values excluding outliers (>1.5 times the interquartile range beyond the box).

| method chain length^a^ | | mean scRMSD^b^ | mean mutual  TM-score | mean number of clashed  residue per backbone^b^ | mean number of geometry  distorsion per backbone^c^ |
| --- | --- | --- | --- | --- | --- |
| PVQD | 70 | 2.95 | 0.32 | 4.71 | 5.71 |
|  | 100 | 2.55 | 0.32 | 6.58 | 10.01 |
|  | 200 | 5.45 | 0.28 | 17.59 | 15.05 |
|  | 400 | 7.18 | 0.27 | 34.37 | 20.09 |
| PVQD+  SCUBA-D | 70 | 1.61 | 0.33 | 1.79 | 3.96 |
|  | 100 | 1.76 | 0.32 | 3.22 | 5.78 |
|  | 200 | 4.67 | 0.28 | 5.83 | 8.70 |
|  | 400 | 8.90 | 0.26 | 17.44 | 10.71 |
| SCUBA-D | 70 | 2.82 | 0.43 | 1.21 | 2.54 |
|  | 100 | 0.78 | 0.39 | 2.37 | 2.98 |
|  | 200 | 2.47 | 0.35 | 7.52 | 8.81 |
|  | 400 | 5.57 | 0.30 | 6.93 | 8.00 |
| Chroma | 70 | 2.26 | 0.38 | 0.45 | 1.03 |
|  | 100 | 3.03 | 0.35 | 0.41 | 1.09 |
|  | 200 | 4.57 | 0.34 | 0.35 | 1.11 |
|  | 400 | 12.41 | 0.30 | 0.45 | 1.06 |
| RFdiffusion | 70 | 1.01 | 0.47 | 0.75 | 1.34 |
|  | 100 | 0.72 | 0.40 | 1.53 | 1.48 |
|  | 200 | 1.21 | 0.35 | 6.91 | 3.25 |
|  | 400 | 6.06 | 0.34 | 19.83 | 7.16 |

**Table S1. Comparative performance of different methods in unconditional backbone generation.**

^a^ For each chain length of 70 residues, 100 residues, 200 residues, and 400 residues, 100 backbones were unconditionally generated with each of PVQD, PVQD+SCUBA-D, SCUBA-D, Chroma, and RFdiffusion.

^b^ Clashes were evaluated by checking all interatomic distances against a sum of atomic radii plus a tolerance factor of 1.5 Å.

^c^ Geometry distortion were evaluated by checking all omega angle against 180^◦^ or 0^◦^ plus a tolerance factor of ± 5^◦^.

**Table S2. Time cost (seconds spent with a single NVIDIA 3090 GPU) of PVQD in unconditional backbone generation and structure prediction.**

| protein length | <100 | 100-200 | 200-300 | 300-400 |
| --- | --- | --- | --- | --- |
| unconditional generation^a^ | 31 | 33 | 36 | 38 |
| structure prediction^b^ | 60 | 63 | 67 | 71 |

^a^ Averaged over 10 independent runs for generating protein structures of 4 different lengths: 50, 150, 250, and 350 residues.

^b^ Structure prediction of 4 native proteins of different lengths: 8FWJ (91 residues), 1CFC (148 residues), 1RPJ (288 residues), and 3T1P (371 residues).

References

1. J. Jumper *et al.*, Highly accurate protein structure prediction with AlphaFold. *Nature* **596**, 583-589 (2021).

2. Y. Zhang, J. Skolnick, TM-align: a protein structure alignment algorithm based on the TM-score. *Nucleic acids research* **33**, 2302-2309 (2005).

3. Z. Lin *et al.*, Evolutionary-scale prediction of atomic-level protein structure with a language model. *Science* **379**, 1123-1130 (2023).

4. J. Dauparas *et al.*, Robust deep learning-based protein sequence design using ProteinMPNN. *Science* **378**, 49-56 (2022).

5. M. van Kempen *et al.*, Fast and accurate protein structure search with Foldseek. *Nature Biotechnology*, 1-4 (2023).

6. Z.-Z. Yu *et al.*, DomBpred: protein domain boundary prediction based on domain-residue clustering using inter-residue distance. *IEEE/ACM Transactions on Computational Biology and Bioinformatics* **20**, 912-922 (2022).

7. J. L. Watson *et al.*, De novo design of protein structure and function with RFdiffusion. *Nature* **620**, 1089-1100 (2023).

8. N. Anand, T. Achim, Protein structure and sequence generation with equivariant denoising diffusion probabilistic models. *arXiv preprint arXiv:2205.15019*, (2022).
